# Separable cryo-microneedle patches delivery with capsaicin integrated mesoporous dopamine for obesity treatment

**DOI:** 10.1186/s12951-025-03645-y

**Published:** 2025-09-03

**Authors:** Jingjing Gan, Lingyu Sun, Wenjuan Tang, Yuanjin Zhao, Yan Bi

**Affiliations:** 1 Department of Endocrinology, Medical School, Nanjing Drum Tower Hospital, Nanjing University, Nanjing, 210002 China; 2 State Key Laboratory of Bioelectronics, School of Biological Science and Medical Engineering, Southeast University, Nanjing, 210096 China

**Keywords:** Separable, Cryo-microneedle, Obesity, Photothermal, Dopamine, Capsaicin

## Abstract

Microneedle patch encapsulated with active medication holds significant potential promise in the realm of anti-obesity therapy. Nevertheless, the improvement of actives delivery efficiency remains a significant challenge. In this paper, we present novel separable cryo-microneedles patches delivered with capsaicin integrated mesoporous dopamine (mPDA) for obesity treatment through activating TRPV1 and inducing lipid droplet dissolution. Such patch is fabricated by cryogenic molding of biocompatible hydrogel tips, which can be easily inserted and left from skin through the thermoresponsive backing layer. Upon penetration, these tips could release mPDA-carrying capsaicin into the targeted subcutaneous adipose layer. In addition, when exposed to near infrared (NIR) light, these mPDA could rapidly convert the light energy into heat to increase the local temperature, thus accelerating the release of capsaicin from mPDA and the metabolic benefits of regional hyperthermia therapy. Based on these features, we have demonstrated that the multifunctional separated cryo-microneedles could significantly reduce weight and relieve obesity-associated pathological conditions with no adverse effect through an 8-week observation on high-fat diet (HFD) mice. Thus, we believe that our separable cryo-microneedles patches delivered with capsaicin integrated mPDA will open a new prospect for obesity treatment.

**Graphical abstract:**

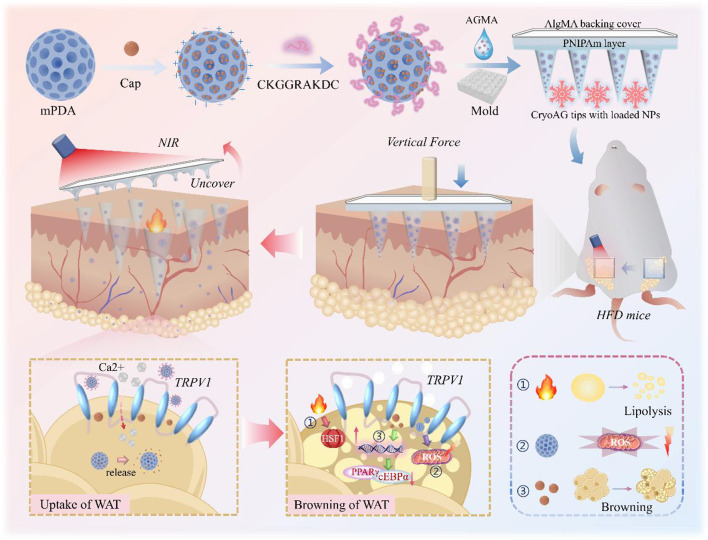

**Supplementary Information:**

The online version contains supplementary material available at 10.1186/s12951-025-03645-y.

## Introduction

Obesity and overweight issues that greatly elevate the risk of metabolic diseases perplex 40% of the global population [1–4]. As an active component in red pepper, capsaicin (Cap) has shown positive effect to combat obesity through regulating lipid metabolism and triggering the browning of white adipose tissue [5–8]. Recent insights suggest that the microneedle patch is a minimally invasive strategy for capsaicin administration to reduce topical drug use and mitigate skin irritation [9–12]. Despite the progress in microneedle-based delivery systems, the majority of hydrogel composed microneedle patches usually lack inadequate mechanical strength to effectively puncture the skin barrier and enter adipose tissue for anti-obesity treatments [13, 14]. In addition, the existing microneedle patches should be attached to treatment sites for a relatively long term, which may bring about inconvenience and discomfort to patients [15–17]. Furthermore, the substantial hydrophobicity and extremely low bioavailability of naked capsaicin hinder its efficient uptake by adipocytes, impeding its therapeutic effect and broader applications [18]. Thus, further efforts to construct new capsaicin-based patches with safe and effective properties are still anticipated.

**Fig. 1:**
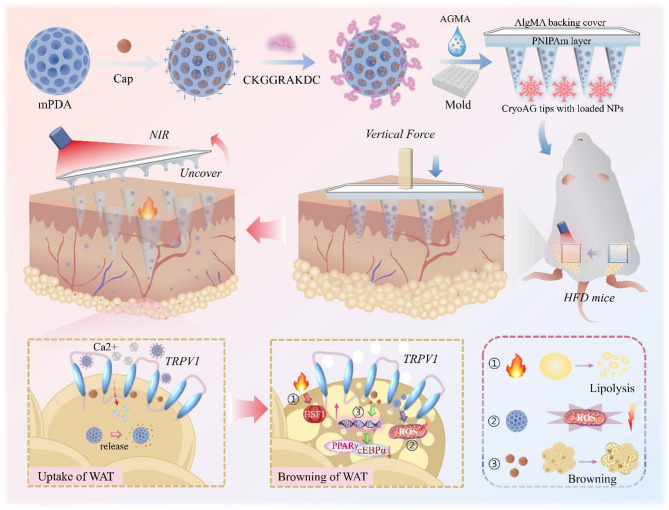
Schematic description of the design and strategy to generate a separable cryo-microneedle for resisting high-fat diet (HFD)-induced obesity

Herein, we proposed novel separable cryo-microneedles patches delivered with capsaicin integrated mesoporous dopamine (mPDA) for obesity treatment through activating transient receptor potential vanilloid-1 (TRPV1) and inducing lipid droplet dissolution, as outlined in Fig. 1. Cryogenic technology offers a positive choice for achieving rapid setting and easy demolding of certain materials (e.g. hydrogels and cryogenic media) [19–21]. The combination of such technique with microneedles can not only impart them with enough mechanical strength to pierce the skin and deliver bioactive molecules for treatment [22, 23], but also endow the separation of microneedle tips through appropriate structure or component design [24]. In contrast, mPDA nanoparticles with mesoporous structure and high surface areas have attracted increasing interest in the field of nano-delivery platforms [25–27]. The abundant functional groups (e.g., catechol and amine groups) on the mPDA surface enable strong π–π stacking and hydrogen bonding interactions with capsaicin molecules, thus enhancing loading efficiency and enabling sustained release [28]. In addition, these mPDA delivery systems could also realize photothermal-controlled drug release and enhance mitochondrial anti-oxidative protection [28, 29]. Thus, we conceive that the integration of capsaicin-encapsulated mPDA into cryo-microneedles could construct a hierarchical separable system to serve as a potential obesity treatment approach.

In this study, we prepared the desired functional microneedle patch through the combination of nanoemulsion assembly, multi-layer replication and freezing strategies. The microneedle patch consisted of biocompatible hydrogel tips with drug loading, a thermosensitive poly-N-isopropylacrylamide (PNIPAm) lift-off layer, as well as an alginate methacryloyl hydrogel-composed backing layer. As the encapsulated capsaicin-loaded mPDA nanocarriers in microneedle tips were modified by adipose-specific peptide, this imparted them with photo-thermally controllable drug release and targeting capacity towards white adipose tissue. After freezing and being applied to the skin, the backing layer would dissolve within several minutes under the effect of body temperature, with microneedle tips left inside the subcutaneous tissue. When exposed to near infrared (NIR) light, mPDA could rapidly convert the light energy into heat to increase the local temperature, thus accelerating the release of capsaicin from mPDA and the metabolic benefits of regional hyperthermia therapy [30]. We have demonstrated that in the high-fat-induced mouse models of obesity, the separated microneedle tips could efficiently facilitate lipolysis and consume the body’s energy via the action of TRPV1 through 8 weeks, leading to increased mitochondria and UCP1 levels, as well as the ultimate reduction of host body weight. These results indicated that our photothermal cryo-patch platform provides a new prospect for obesity treatment.

## Results

### Preparation and characterization of TmCNP

**Fig. 2:**
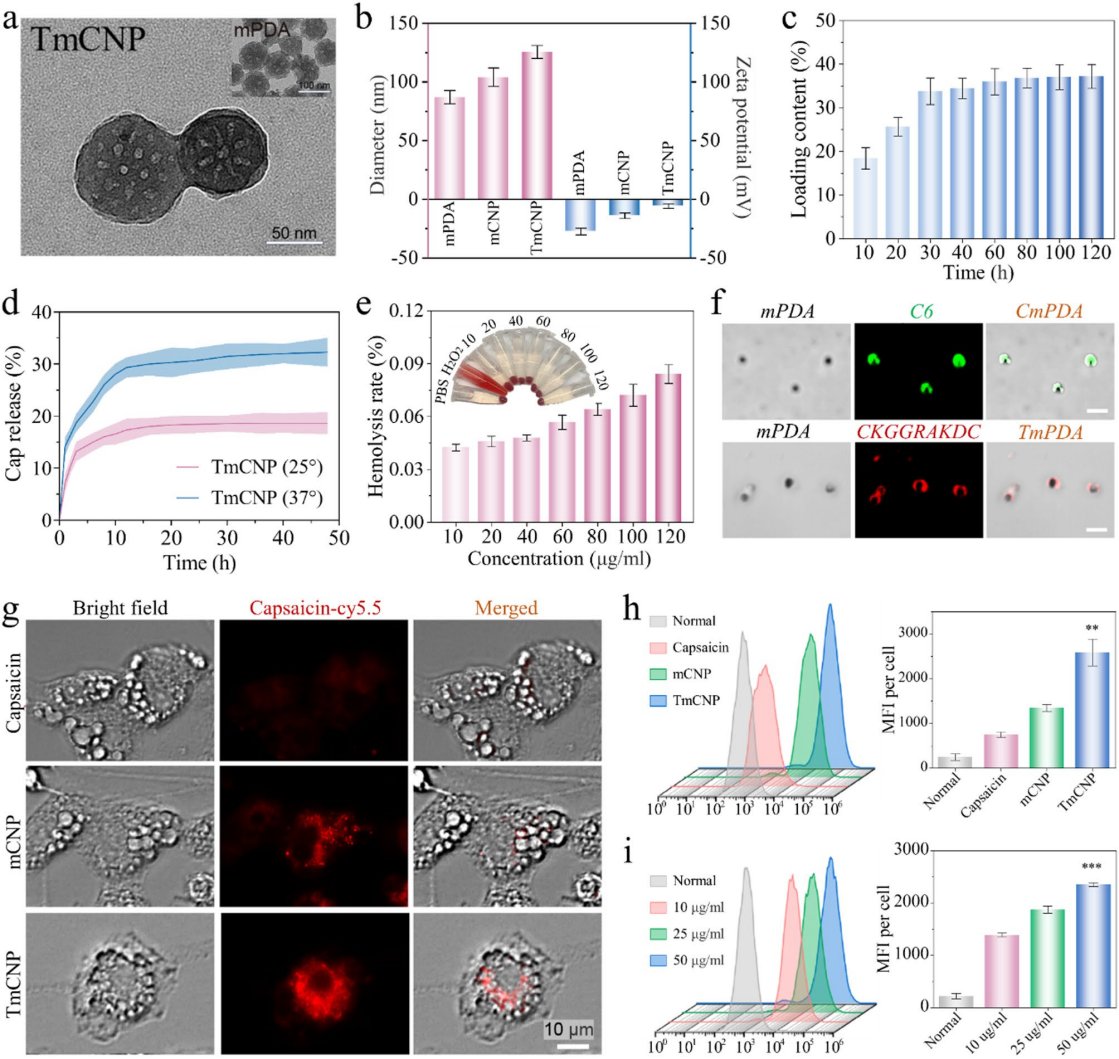
Characteristics and in vitro transfection of TmCNP. (**a**) TEM images of TmCNP and mPDA nanosphere. (**b**) Average size and charge of mPDA, mCNP (mPDA-loaded Cap), and TmCNP. (**c**) Influence of incubation time on capsaicin loading content, determined by measuring the free drug content in TmCNP. (**d**) Cumulative release curves for TmCNP at 25 ℃, 37 ℃, and 42 ℃. (**e**) Hemolysis assay results at varying concentrations of TmCNP after centrifugation. (**f**) Photographic representation of mPDA, C6 (represented Cap, green), and CKGGRAKDC (marked with cy5.5, red). Scale bar, 100 nm. (**g**) Representative cellular uptake images of cy5.5-labeled Cap (10 µg/ml), mCNP (mPDA&cy5.5-labeled Cap, containing an equivalent amount of cy5.5-labelled Cap at 10 µg/ml), and TmCNP (TmPDA&cy5.5-labeled Cap, also equivalent to 10 µg/ml cy5.5-labled Cap) in mature adipocytes after 6 h of incubation at 37 ℃. Scale bar, 10 μm. (**h**) Representative flow cytometric histograms showing cellular uptake of cy5.5-labeled nanoparticles (Cap, mCNP, and TmCNP with equivalent cy5.5-labeled Cap concentration at 10 µg/ml) in mature adipocytes differentiated from 3T3-L1 preadipocytes. Mean fluorescence intensity (MFI) for cy5.5-labeled Cap per preadipocyte. (**i**) Flow cytometry plots and quantification of the MFI at different concentrations of TmCNP (cy5.5: 5, 10, 30 µg/ml). Normal vs. TmCP **p < 0.01, Normal vs. 30 µg/ml ***p < 0.001, determined by one-way ANOVA in (**h-i**)

Given the known capacity of mPDA to store drugs and scavenge excessive free radicals, we synthesized uniform mPDA with a mean diameter of about 87.2 nm and a pore size of around 8 nm using the nanoemulsion assembly method (Supplementary Fig. 1). Sequentially adding Cap and peptide CKGGRAKDC in phosphate-buffered saline (PBS, pH7.4), the generated adipocyte-targeting nanodrugs (TmCNP) were characterized using transmission electron microscope (TEM) and a Zetasizer analyzer (Fig. 2a-b). The results indicated that mPDA was wrapped with a thin peptide layer on its surface and the shape, size and surface charge of TmCNP were only slight affected by agent loading and modification. The drug loading performance of such carrier reached saturation within a short time (Fig. 2c). In vitro release testing illustrated that a higher release at 37 ℃, with a c

umulative release of 29.5% within 12 h, suggesting that warming significantly accelerated this process (Fig. 2d**)**. The drug release behavior of TmCNP under NIR laser irradiation was investigated, demonstrating that repeated laser exposure in PBS resulted in a photothermal effect-driven release of 40% over 10 h, likely due to heat-induced acceleration of drug molecule diffusion (Supplementary Fig. 2). The formed nanospheres supported the good blood compatibility, as indicated by hemolytic activity (Fig. 2e). Thus, TmCNP appears to be a safe and effective vehicle for drug encapsulation and delivery.

We then validated the affinity of TmCNP for mature adipocytes before bioactivity detection. By employing hydrophobic Coumarin 6 (C6) instead of Cap and cy5.5 labeled CKGGRAKDC, we observed a clean green signal around the mPDA particles, with the drug-to-carrier ratio well controlled (Fig. 2f). Additionally, the particles were able to enrich CKGGRAKDC on their surface, presenting a red fluorescence morphology (cy5.5 labeling). As anticipated, TmCNP was able to specifically penetrate mature adipose cells (Fig. 2g), with uptake increasing dose-dependently, as shown by flow cytometry and quantitative statistics (Fig. 2h-i). In contrast, only minimal red fluorescence was observed within preadipocyte samples (Supplementary Fig. 3a). Flow cytometry data further confirmed that the uptake of TmCNP by adipocytes was significantly higher than that by 3T3-L1 cells (Supplementary Fig. 3b). In summary, these data underscore the targeting capability of TmCNP, which is essential for the efficient delivery of Cap into white adipose tissue.

### Anti-Lipogenesis and pro-browning effects of TmCNP

We aimed to evaluate whether the bioactivities of TmCNP may inhibit lipogenesis and promote adipocyte browning in vitro. The biocompatibility characteristics of the TmCNP were initially investigated using the 3T3-L1 cell line, as shown in Supplementary Fig. 4, which confirmed the absence of cytotoxicity.

**Fig. 3:**
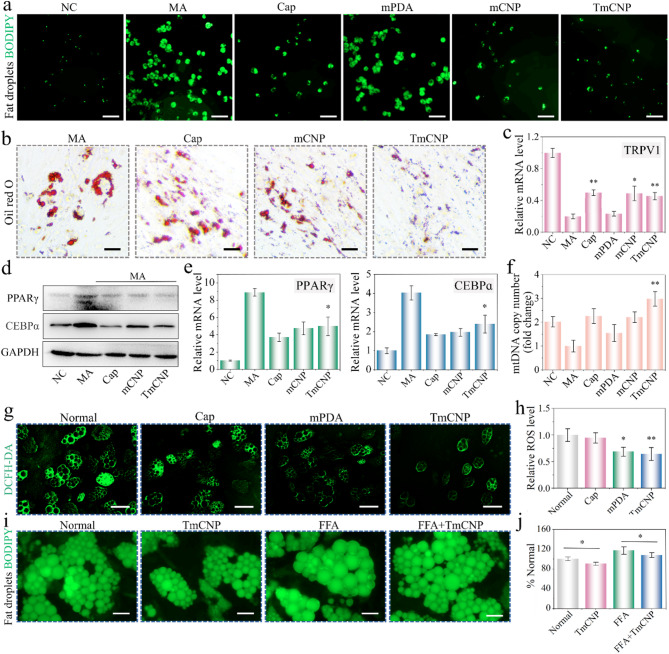
Adipocyte browning, anti-lipogenesis, and anti-oxidant effects of Cap-loaded mPDA on adipocytes. **(a)** Representative BODIPY-stained images of differentiated adipocytes. Pre-adipocytes were induced to differentiate for 8 days into mature adipocytes (MA), followed by treatment with Cap, mPDA, mCNP, or TmCNP, for 48 h. NC: undifferentiated ADSCs. Scale bar: 200 μm. **(b)** Oil red O staining photographs of adipocytes treated with different conditions. Scale bar, 100 μm. **(c)** Q-PCR analysis of TRPV1 mRNA levels in mature adipocytes treated with Cap, mPDA, mCNP, and TmCNP. **(d)** Western blotting analysis was performed to assess PPARγ and CEBPɑ protein levels in cells following various treatments. **(e)** Representative mRNA quantification of PPARγ and CEBPɑ in adipocytes subjected to various treatments. **(f)** Results of mtDNA copy number of untreated preadipocytes, mature adipocytes and MA treated with Cap, mPDA, mCNP, or TmCNP. **(g-h)** ROS levels in adipocytes after different treatments following pre-induction with palmitic acid in 0.5 mM for 24 h. **(i)** Representative confocal images of mouse adipocytes stimulated with palmitic acid. Cells were labeled with BODIPY dye following treatment with TmCNP. **(j)** Cellular TG content was detected and normalized to intracellular total protein content. Normal: MA, *p < 0.05, **p < 0.01

To evaluate the anti-lipogenesis function, we used the primary mouse adipose-derived stem cells (ADSCs) of 8-day differentiation as mature adipocyte model [31]. Compared to cell lines, ADSCs offer a more biologically relevant system that better mimics native adipose tissue. BODIPY staining revealed a significant accumulation of lipid droplets in the successfully differentiated adipocytes. Compared with the free Cap group, the mPDA-treated group had a weak effect in reducing lipid droplet accumulation, while the TmCNP-treated group showed a significant reduction in lipid droplets (Fig. 3a). These findings were further corroborated by Oil red O staining (Fig. 3b). Compared to untreated mature adipocytes, mRNA and protein expression of TRPV1 (the known Cap receptor [32]) was significantly upregulated in cells treated with Cap, mCNP, or TmCNP (Fig. 3c and Supplementary Fig. 5). Next, by using the Q-PCR and Western blot assays, we observed a significant downregulation of both protein and mRNA levels of PPARγ and cEBPα in differentiated adipocytes following continuous treatment with TmCNP during the differentiation process (Fig. 3d and e). We also evaluated the upregulation of mRNA levels of browning and mitochondria biogenesis-related gene makers [33], including UCP1, PGC-1α, and Cyto C, in comparison with the blank group (Supplementary Fig. 6a). Measurement of lipolysis markers [34] – lipoprotein lipase (LPL) and adipose triacylglyceride lipase (ATGL) – demonstrated that the increase in lipolytic activity of Cap group was less significant compared to the TmCNP group (Supplementary Fig. 6b).

To understand more clearly the changes in mitochondrial function, we further examined the copy number of mtDNA following the different treatment via a quantification Q-PCR assay kit. As expected, the mtDNA copy number in total adipocyte with TmCNP was much higher than that in the other samples, indicating a stronger effect in promoting mitochondrial biogenesis (Fig. 3f). Mature adipocytes contain large amounts of lipids that are readily oxidized by reactive oxygen species (ROS), resulting in the formation of metabolic byproducts. Moreover, adipocyte differentiation produces a substantial amount of ROS [35]. Since we observed a significant reduction in intracellular ROS in adipocytes treated with either mPDA or TmCNP, fluorescence probe DCFH-DA staining was performed followed by quantification. The data consistently highlighted the antioxidant capability of mPDA (Fig. 3g-h). To further evaluate the effects on adipogenesis, palmitate was applied to exacerbate adipocyte hypertrophy through triglyceride (TG) deposition (Supplementary Fig. 7), following various treatments as visualized by BODIPY staining. As expected, TmCNP treatment significantly reduced the lipid droplet content (Fig. 3i). Similarly, TmCNP greatly decreased intracellular TG level in response to palmitate stimulation, as determined by glycerol assay kit (Fig. 3j).

### Design of the cryogenic AGMN for delivering photothermal nanodrugs

To build a patch composed of tip-loading drugs and separable backing cover, the first step is to devise a microneedle matrix with two key elements. First, the tip is a hard enough to pierce the skin; second, it should provide a mechanism to persistently release the TmCNP to promote browning and energy consumption in white adipose tissue (WAT).

**Fig. 4:**
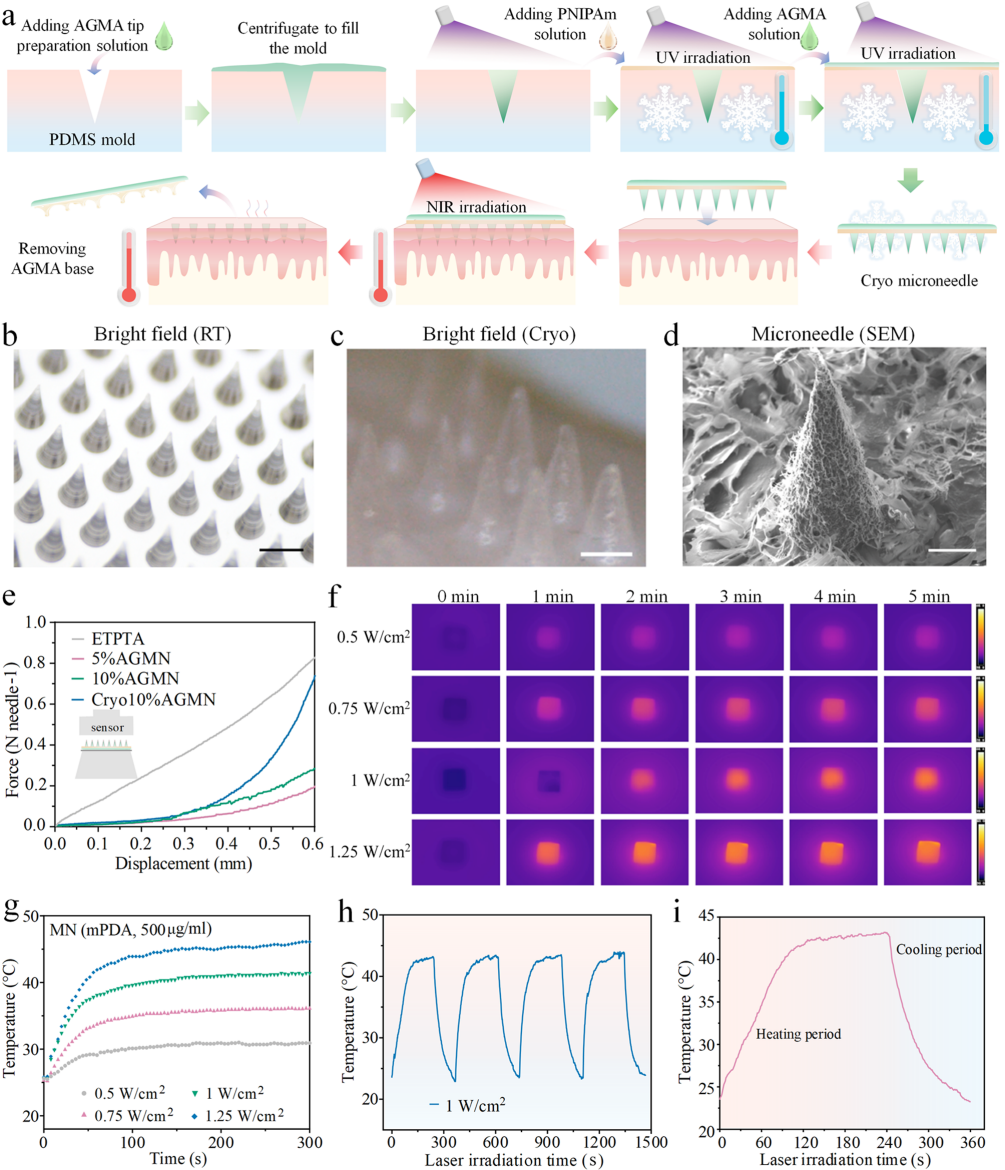
Schematic illustration and characterization of AGMN prepared through a freezing process. (**a**) A schematic of the patch fabrication process. (**b**) Bright-field visualization of the AGMN patch at room temperature. Scale bar, 1000 μm. (**c**) Digital picture of the cryo-MN. Scale bar: 300 μm. (**d**) SEM photograph of patch tip. Scale bar, 200 μm. (**e**) Mechanical testing of the MNs. (**f**) Photothermal pictures and (**g**) temperature heating curves of AGMNs loaded with TmCNP (mPDA concentration: 500 µg/ml) under varying NIR intensities. (**h**) Results of the photothermal stability of AGMN over 4 cycles of NIR irradiation. (**i**) Temperature variation assay of AGMN@TmCNP during heating and cooling phases

Then, we developed the separable patch to deliver Cap-laden mPDA to intradermal layer for effective WAT browning (Fig. 4a). To establish the tip, we employed a AlgMA-mixtured GelMA (AG) as the building block of the microneedle tip for skin penetration. Adding AlgMA-GelMA solution into mold and then cured under UV to form the AGMA structure. The thermosresponsive PNIPAM polymer was positioned between the backing layer (top part, base) and the AGMA tip. PNIPAm is a thermoresponsive polymer that has the ability to change its solubility and hydrophilicity within a specific temperature range [36–38]. This property enables the easy separation of the microneedle backing layer from the skin, leaving the microneedles embedded within the skin. The resulting AGMN consisted of 10 × 10 arrangement with height and base weight of 1000 μm and 380 μm, respectively (Fig. 4b). And AGMN patch was solidified after cryogenic process (Fig. 4c). As tip must be sharp enough to penetrate the skin, the AG tip can be molded in a cryogenic state that have strong mechanical properties. The solidified AG tip presented a porous microstructure as revealed by SEM (Fig. 4d), with an average pore size of 325 ± 21.3 nm that is desirable for nanosphere loading. We selected PINPAM hydrogel supplemented with Lithium phenyl(2,4,6-trimethylbenzoyl) phosphinate (LAP) to fabricate the separating layer of backing (Supplementary Fig. 8a). Then AlgMA gel was placed onto the separating framework for peeling patch base from the dermis layer (Supplementary Fig. 8b). SEM images of microneedles before and after penetration into agarose gel demonstrated that the presence of PINPAM layer successfully separated the back and leave the tip under the skin (Supplementary Fig. 9). Typical fluorescence images of AGMN patch confirmed the successful assembly of components (Supplementary Fig. 10). Furthermore, we evaluated the mechanical strength of the cryo-MNs through a tensile tester. The results indicated that the breaking load of the patch was 0.57 N (Fig. 4e), significantly exceeding the minimum average force of 0.058 N required for skin penetration [39].

To understand more clearly the photothermal properties of AGMN under NIR laser radiation, we captured the photothermal images and recorded heating curves of AGMN mixed with mPDA (500 µg/ml) at irradiation power densities of 0.5, 0.75, 1 and 1.25 W/cm^2^ over a duration of 5 min (Fig. 4f-g). Besides, different concentrations of mPDA were assessed over 5 min, as illustrated in Supplementary Fig. 11–12. The heating curves of patch containing mPDA nanomaterial revealed that mPDA exhibited photothermal heating performance dependent on both concentration and power density (Fig. 4g). Since the body temperature cannot exceed 42 °C for extended periods, we selected microneedles containing 500 µg/ml of mPDA for in vivo experiments under 1 W/cm² infrared irradiation. It was found that the selected patch under 1 W/cm^2^ exhibited strong photothermal stability. Next, the photothermal stability of AGMN was demonstrated by using four consecutive irradiation ON/OFF cycles (Fig. 4h-i). For cytotoxicity measurement, it was found that the material exhibited negligible cytotoxicity, with cell viability remaining above 90%, indicating good biocompatibility for biomedical applications (Supplementary Fig. 13). Result for in vivo degradation of the AG hydrogel indicated that the material underwent gradual and controlled degradation in subcutaneous tissue (Supplementary Fig. 14).

### TmCNP released from NIR light irradiated AGMN in a mouse model

**Fig. 5:**
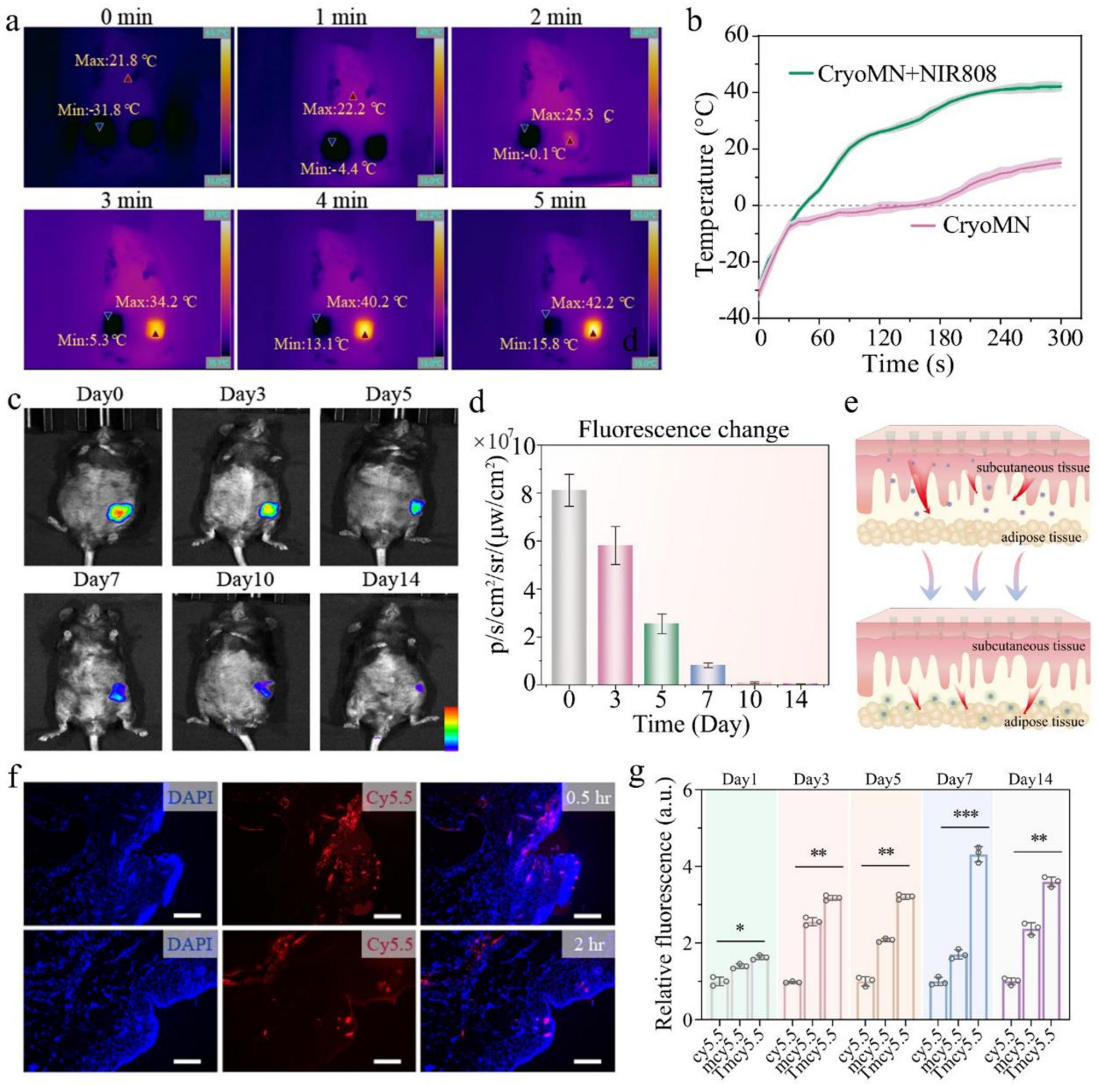
The skin penetration capacity, photothermal property and release of nanodrugs of the AGMN. (**a**) Thermal imaging pictures of animals applying AGMN to the left skin of the inguinal region at the specified time points. (**b**) Temperature change profiles of left skin of the MN-penetrated location under laser irradiation. (**c**) Fluorescence biodistribution in HFD-induced mice appied with cy5.5-labeled MNs on day 3, 5, 7, 10, and 14. (**d**) Results of quantitative analysis of fluorescence intensity in the skin areas penetrated by the patch. (**e**) Schematic of sustained-releasing AGMNs from skin to adipose tissues. (**f**) Typical images from skin fluorescence sections following treatment of cy5.5-mPDA-loaded AGMNs. Scale bar: 500 μm. (**g**) Flow cytometric quantification results for isolated adipocytes labeled with cy5.5

An infrared thermal imager was employed to record the temperature distribution of the microneedles on both a table and human fingers. The results indicated that the microneedles on the fingers rapidly returned to room temperature, highlighting the need for immediate insertion of the frozen microneedles into skin tissue (Supplementary Fig. 15). Next, we pierced the AGMN under NIR light exposure in mice subjected to an 8-weeks the high-fat diet (HFD) to examine the photothermal and release property of nano-vehicles. As such, a thermal imaging system was employed to monitor subcutaneous temperature variations during the application of AGMN. Since mPDA is a typical photothermal material [40], we measured the patch working area in the inguinal WAT region and plotted the thermography. It showed that the local temperature where NIR light applied was markedly higher than the other parts, with a temperature increase from − 31.8 to 42.2 °C after 5 min of treatment (Fig. 5a). Such photothermal phenomenon did not occur in the control group, as confirmed by the quantification data (Fig. 5b). We then utilized in vivo imaging equipment to monitor the biodistribution of cy5.5-marked TmCNP during the application of AGMN. Upon microneedle application, cy5.5 was visible at subcutaneous white adipose tissue (scWAT) for 7 days in HFD model, consistent with quantitative trends observed (Fig. 5c-d). The TmCNPs encapsulated in this patch could release into the subcutaneous WAT layer, as illustrated in Fig. 5e. The subcutaneous tissues beneath the patch application were harvested and immediately examined, revealing strong red fluorescent signals in frozen section of scWAT tissues at 0.5 h. Moreover, the nanocarriers rapidly entered subcutaneous tissues within 2 h, indicating effective drug delivery via the patch system (Fig. 5f). To verify fat targeting in vivo, we conducted flow cytometric analysis of cy5.5-labeled adipocytes after treatments at 1, 3, 5, 7, and 14 days following the same AGMN applications. The experimental data indicated that encapsulation and delivery of TmCNP via microneedle can synergistically facilitate the release and targeting of Cap into scWAT (Fig. 5g).

To investigate the safety associated with in vivo therapy, we assessed the histological change in skin tissue and various organs from both untreated and those treated with cryo-AGMN. The H&E analysis results indicated that cryo-AGMN treatment caused no significant toxicity to the skin tissue or major organ tissues (Supplementary Fig. 16). Additionally, histopathological analysis of skin tissue punctured by cryo-MNs for 0.5 and 24 h confirmed that the low temperature did not cause tissue damage (Supplementary Fig. 17). The proportions of CD3⁺ and CD45⁺ cells in mouse skin were marked one-week post-application to monitor potential immune responses (Supplementary Fig. 18a). Moreover, skin samples from the application site were histologically analyzed after successive applications (Supplementary Fig. 18b). The results revealed no evidence of inflammation or tissue damage, indicating that the patch possesses good biocompatibility.

### NIR&AGMN@TmCNP reduces obesity in a mouse model

Given the findings that AGMN can penetrate the skin of obese mouse, we further explored its potential for addressing obesity using an HFD model, as diet-induced obesity in animals is comparable to obesity in humans [41, 42].

**Fig. 6:**
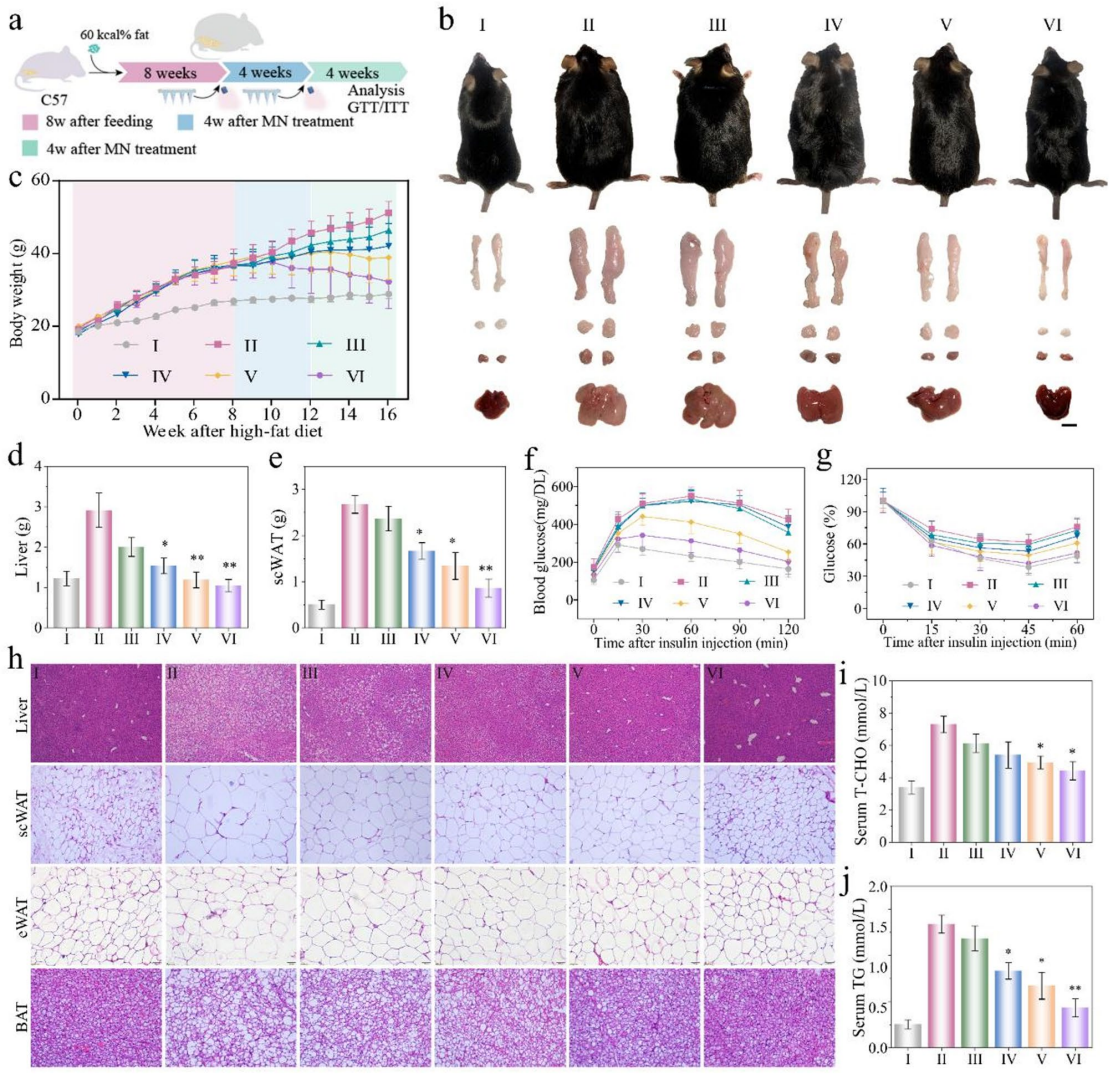
Therapeutic efficacy of NIR@MN in HFD animal. (**a**) Schematic diagram of the experimental process of microneedle treatment of obesity. After 8 weeks of HFD, mice began to receive microneedle treatment. During the treatment, the mice were anesthetized, the abdominal hair was shaved, and the microneedles were inserted into the skin of the left and right abdomens. After pressing for 2 min, the back was uncovered and infrared irradiation was performed for 5 min (1 W/cm^2^). After that, infrared irradiation was performed once every three days, and the above treatment was performed again after 4 weeks. At the end of the experiment after 8 weeks MN treatment, the animals were sacrificed, specimines were harvested for subsequent examination. (**b**) Photographs depict-induced obese mice, along with their adipose tissues (scWAT, eWAT, and BAT) and livers, following 56 days of the various treatments. (**c**) Weekly body weight measurements (*n* = 6 per group). (**d-e**) Weights of the liver and scWAT; (**f**,** g**) GTT performed 6 weeks post AGMN implantation and ITT performed 10 weeks post AGMN implantation of the animals. (**h**) H&E stained liver, scWAT, eWAT, and BAT photographs; (**i**,** j**) Serum total lipid contents including total TCHO and TG. **p < 0.01; *p < 0.05, between AGMNs-treated mice on HFD and untreated mice on HFD. Refer to the experimental methods section for detailed grouping information

In the obese model, increased fasting blood glucose, body fat accumulation and insulin resistance began to appear after 8 weeks of HFD induction. To assess the resistive effects of AGMN, we applied two cryo-MNs to the abdomen area of the model mice (Fig. 6a). The NIR&AGMN mice gained weight much slower than the control animals 8 weeks post-penetration (Fig. 6b). The body weight of NIR&MN-treated mice was markedly lower than of control mice (average NIR&MN 36.4 g versus control group 48.56 g) (Fig. 6c). Notably, the liver and scWAT beneath the application site of patch were obviously smaller and lighter than the control group; this outcome is consistent with the statistical quantification in Fig. 6d-e. Evaluation results of intraperitoneal glucose tolerance test (GTT) and the insulin tolerance test (ITT) showed that microneedles under near-infrared irradiation could significantly alleviate HFD-induced insulin resistance (Fig. 6f-g). By resisting obesity, NIR combined with patch effectively inhibited the development of hepatic steatosis, as evidenced by reduced liver lipid accumulation in H&E staining results (Fig. 6h) and lower levels of the associated biochemical parameters (Supplementary Fig. 19). The H&E pathological sections highlighted a notable shrinkage of scWAT, epididymal white adipose tissue (eWAT), and brown adipose tissue (BAT) in NIR&MN-treated mice (Fig. 6h), consistent with the statistics (Supplementary Fig. 20). Additionally, the volume of adipocytes isolated from scWAT were significantly decreased in the mice with NIR&MN than in the control animals (**Supplementary Fig. 21**). A lower level of TG and total cholesterol (TCHO) in the serum of the NIR&MN mice further confirmed the significant anti-obesity effect observed. This difference was confirmed by the quantification data but did not occur to the control group (Fig. 6i-j).

### Physiological effects on NIR&MN treatment on HFD-induce mice

**Fig. 7:**
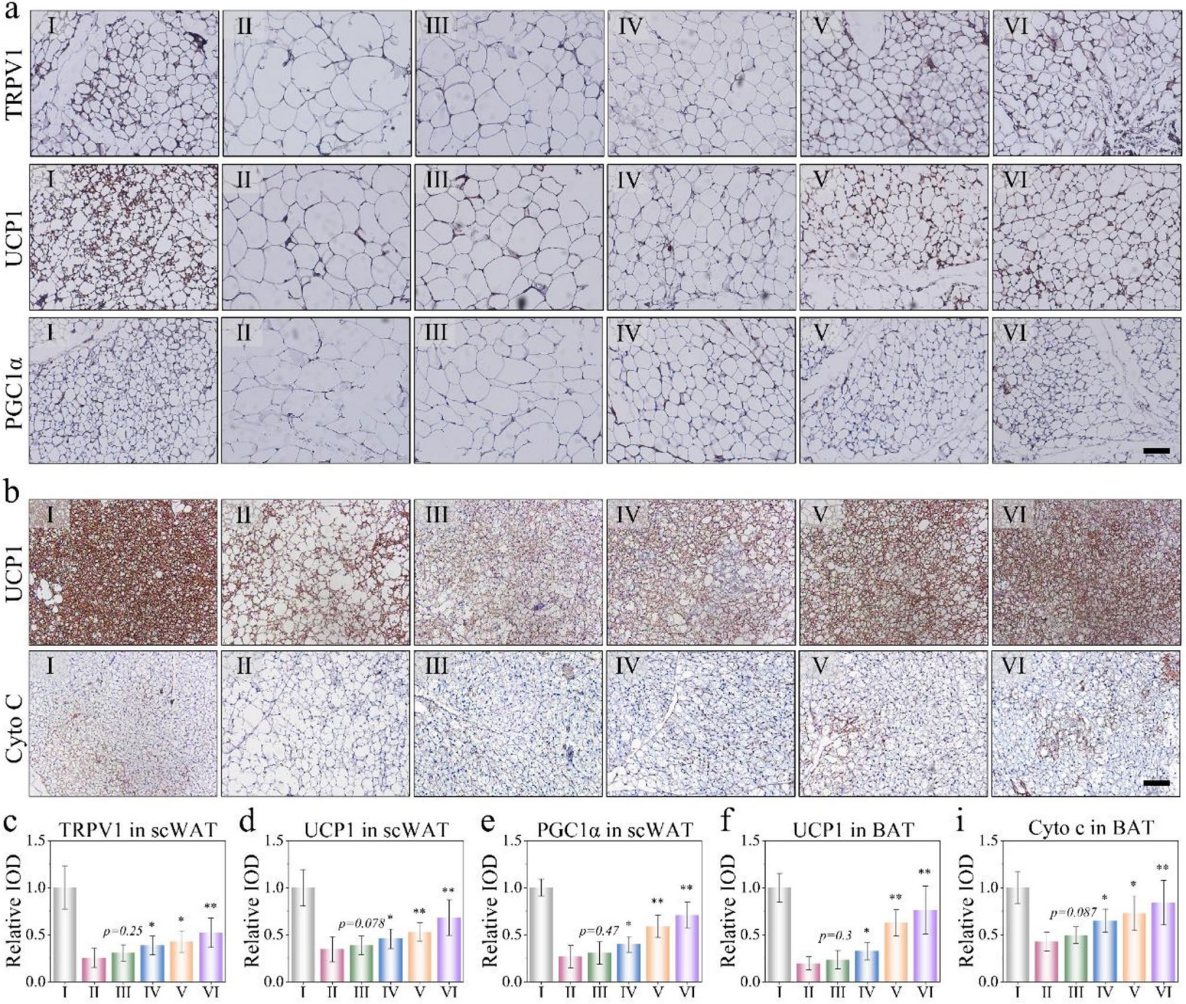
Physiological impacts of the NIR&AGMNs carried TmCNP on obese animals. **(a)** Sections of the adipose immunostaining against the TRPV1, UCP1, and PGC1α protein in scWAT. Scale bar, 50 μm. **(b)** Immumohistochemical staining for UCP1 and Cyto C in BAT. **(c-i)** Quantification of IHC results in (**a-b**). *p < 0.05; **p < 0.01, refer to the experimental methods section for detailed grouping information. *n* = 5 ~ 7 mice per group

Having established the anti-obesity efficacy of the patch in the obesity model, we next investigated the mechanism of NIR&MN therapy in greater detail. We subsequently detected the protein levels associated with browning and mitochondria biogenesis in scWAT and BAT from the differently administrated animals using immunohistochemistry. Firstly, it was observed that the brown signal for TRPV1 protein in NIR&MN treated group significantly increased compared to the untreated obese sample (Fig. 7a), which can be attributed to the function of Cap. Moreover, we found that levels of multiple browning and mitochondrial activity, including PGC1α, UCP1, and Cyto C, significantly upregulated in both scWAT and BAT of AGMN treated obese mice compared to those receiving other groups (Fig. 7a-b), consistent with quantitative data of gray value via image J software (Fig. 7c-i). These results indicated that AGMN&TmCNP can effectively regulate energy metabolism and browning in adipose tissues, with microneedle transdermal administration proving superior to direct subcutaneous injection of Cap. TmCNP appeared to improve mitochondrial activity in BAT associated with obesity, which may be linked to weight loss, reduced BAT fat storage, and enhanced energy metabolism. Furthermore, based on existing literature, we speculated that local thermogenesis caused by infrared-mediated mPDA may also contribute to an anti-adipogenic effect [30], akin to findings from studies on fat reduction using dopamine nanoparticle-based hydrogels.

To further elucidate the mechanistic role of TRPV1, additional in vivo experiments were conducted using the TRPV1 antagonist capsazepine (CPZ), administered intraperitoneally prior to microneedle treatment. Inhibition of TRPV1 significantly attenuated the therapeutic effects, as indicated by impaired lipid clearance in the liver and serum, along with reduced UCP1 expression in scWAT (Supplementary Fig. 22–23). These findings highlighted the critical role of TRPV1 in mediating the observed metabolic improvements. To clarify the role of local hyperthermia, we conducted control experiments using a hyperthermia-only group with NIR exposure. In Supplementary Fig. 24, the HFD group had no effect on fat mass, while the hyperthermia-only group showed slight fat reduction and modest UCP1 upregulation, suggesting limited browning effects from local heating.

## Discussion

In conclusion, we developed a novel separable microneedle patch using s a cryogenic process as a potential approach for anti-obesity therapy. This cryo-patch was specifically designed to enhance energy expenditure and demonstrated both significant efficacy in body weight regulation and favorable safety profile in HFD mice. Capsaicin, a well-known TRPV1 channel agonist, was efficiently loaded into mPDA through adsorption. To achieve adipose tissue specificity, we conjugated the white adipose-targeting peptide CKGGRAKDC to the capsaicin-loaded mPDA, yielding TmCNP nanocarriers capable of precise delivery. These photothermal nanocarriers were further embedded into a UV-curable separable hydrogel microneedle patch, enabling effective and localized drug administration. The frozen insertion of the microneedle tips into the skin caused minimal disruption to tissue integrity or pharmaceutical activity. Mechanistically, capsaicin activated TRPV1 channels, leading to calcium influx, sympathetic nerve stimulation, and WAT thermogenesis, thereby increasing energy expenditure. Simultaneously, mPDA-mediated photothermal conversion under NIR irradiation generated localized mild hyperthermia (~ 42 °C), which further activated TRPV1, amplifying the effects of capsaicin. These findings suggested a synergistic mechanism between chemical and photothermal stimuli in driving metabolic activation. In vivo studies confirmed that the combination of AGMN and TmCNP significantly enhanced systemic energy expenditure and conferred notable therapeutic benefits without safety concerns. This integrated approach holds great potential for treating obesity and associated metabolic comorbidities, including steatohepatitis, hyperlipidemia, type II diabetes, and hypertension.

## Methods

### Material

The dopamine hydrochloride and the pluronic F-127 were obtained from Sigma (St Louis, USA). Capsaicin (≥ 99.47%) was obtained from lemeitian medicine (Chengdu, China). Fluorescent hydrophobic dyes, including cy5.5 and coumarin 6 were derived from yuanye Bio-technology (Shanghai). CKGGRAKDC was obtained from Nanjing Peptide Biotech Ltd. BODIPY fluorescent dye was sourced from MedChemExpress Co., Ltd. The Calcein-AM/PI viability/cytotoxicity kit, reactive oxygen species detection kit, oil red O staining kit, CCK-8 regent, and H&E staining kit were provided by Beyotime Co. Antibodies, including anti-Cyto C, anti-PPARγ, anti-UCP1, anti-cEBPα, and anti-TRPV1 were provided by Abcam Co., Ltd.

### Synthesis and characterization of TmCNP

The mesoporous PDA (mPDA) was synthesized using a versatile nanoemulsion assembly approach. To encapsulate Cap within the mesopores, mPDA and Cap were gently mixed and rotated at a mass ratio of 5:1 and continuously rotated to produce the Cap-loaded mPDA. Then, the unencapsulated Cap was removed by dialysis. An excess of CKGGRAKDC solution was slowly added to the Cap-loaded mPDA system at a mass ratio of 1:10. The solvents were then removed through freeze-drying, yielding a nanosphere powder.

The obtained TmCNP powders were dispersed in ultrapure water (1 mg/ml), and a single drop was placed onto a copper grid, followed by counterstaining with phosphotungstic acid. The shape image of TmCNP was observed using TEM equipment (FEI Talos F200X). The ζ potential and size were assessed using DLS Instruments (Malvern, UK).

### In vitro release kinetics of drug from NPs

C6 was utilized as a substitute for Cap in these experiments. The C6 loading efficiency of mPDA and TmPDA was first measured, with values of approximately 31.0% (538.34 ± 21.76 µg/mg) and 39.37% (649.34 ± 19.76 µg/mg), respectively. 10 mg of TmPDA@C6 were dissolved in 2 ml PBS and placed into a dialysis bag (Mw = 3500 Da), which was subsequently immersed in 10 m of PBS. During dialysis, 0.2 ml samples were collected at predetermined time points, and an equal volume of fresh PBS was added. To investigate the laser-triggered drug release behavior of the nanoparticles, C6-loaded NPs were irradiated with an 808 nm laser at a power density of 1 W/cm² for 5 min at predetermined time points (2, 4, 6, and 8 h). The PBS solution was then collected for fluorescence absorption value to quantify the amount of C6 released. The release of C6 from the NPs was determined using fluorescence microplate analysis.

### Hemolysis experiment

Red blood cells (RBCs) from nice were diluted with PBS to a final concentration of 4%. A 0.5 ml aliquot of the RBC suspension was incubated with an equal volume (0.5 ml) of TmCNP solutions at various concentrations, ranging from 10 to 500 µg/ml, at 37 °C for 1 h. Positive controls (100% hemolysis) were prepared using 0.1% Triton X-100, while negative controls (0% hemolysis) were prepared with PBS. After incubation, the samples were centrifuged at 3200 rpm for 15 min, and the absorbance of the supernatant was measured at 576 nm. The hemolysis rate = (A_sample_-A_negative_)/(A_positive_-A_negative_) × 100%.

### In vitro biocompatibility

The cytotoxicity of NPs and MN materials was evaluated using the CCK-8 assay. Briefly, 3T3-L1 cells in good growth status were harvested and seeded into 96-well cell culture plates (8 × 10^4^ cells/ml), followed by overnight incubation. The cells were then exposure to NPs (10 µg/ml) or to 48-hour MN material extracts in cell culture medium, followed by incubation for the specified duration. The treated 3T3-L1 cells were further analyzed using CCK-8 regent and Calcein-AM/PI staining dye. The results were quantified via a microplate reader and visually assessed using a fluorescence microscope.

### Primary adipocytes study

Abdominal adipose tissue was harvested from 7-day-old mice, minced, and digested in HBSS with 2% BSA and 2 mg/ml collagenase I for 25 min at 37 °C on a shaker (100 rpm). Preadipocytes were then collected by centrifugation at ~ 500 g for 8 min and plated in DMEM/F-12 media supplemented with 10% serum. After cell fusion, adipocyte differentiation medium was introduced, containing 125 nM indomethacin, 5 mM isobutylmethylxanthine, 800 nM insulin, 1 µM rosiglitazone, and 5 µM dexamethasone. On day 2 differentiation, the cells were maintained in media containing 1 nM rosiglitazone and 10 nM insulin, with media changes occurring every other day.

For the cell uptake study, hydrophobic cy5.5 was used as a surrogate for Cap to visualize uptake. Induced adipocytes and 3T3-L1 preadipocytes were treated with TmPDA&cy5.5 (cy5.5: 10 µg/ml) at 37 °C for 6 h. Redundant NPs were removed, and cell nuclei were labeled with DAPI for 8 min. After removing the remaining dye, cellular uptake of the fluorescent nanoparticles was observed and captured under a confocal microscope.

Adipocytes treated with TmCNP for 24 h was collected and lysed for protein analysis (PPARγ and cEBPα). The mRNA levels of intracellularly expressed TRPV1, UCP1, and Cyto C were determined using a reverse transcription kit. Primers applied for Q-PCR assays are listed in Table S1. Lipid droplets were stained with BODIPY green fluorescent dye and oil red O, and visualized via fluorescence microscopy. The mtDNA copy number was determined by Q-PCR, using the nuclear-encoded gene B2M as an internal reference.

For intracellular ROS analysis, the induced adipocytes were treated as indicated and then incubated with fresh media containing 5 µM DCFH-DA (Beyotime Biotechnology) at 37 °C for 25 min. The cells were subsequently washed four times with PBS and captured using confocal microscopy.

### Stimulation of adipocytes with palmitic acids

After 6 days of induction, the culture medium of the adipocytes was replaced with F12/DMEM supplemented with 4% serum and 0.5% fatty acid-free BSA. The cells were then treated with 500 µM palmitic acid (containing 10% BSA) for 4 days. Following this treatment, nanoparticles were applied to evaluate the changes in lipid droplets and triglyceride levels within the cells.

### Fabrication of TmCNP-loaded cryoAGMN

AGMNs were designed and prepared using a PDMS mold. A 200 µl volume of an optimized hydrogel mixture, consisting of 5% (w/v) AlgMA and 5% (w/v) GelMA containing 0.2 mg of TmCNP, was cast into the mold and centrifuged at 3,500 rpm for 4 min to fill the needle cavities. Next, 50 µl of a 5% cold PNIPAM solution containing 2.5% PLA was evenly applied to the flat cavity of the mold and irradiated under UV light for 30 s to form a separable layer. For the back layer, 50 µl of 10 wt% AlgMA solution was uniformly applied on top of the solidified PNIPAM gel. The fabricated patch was then frozen at − 80 °C for 2 h and carefully detached from the mold using adhesive tape.

### Characterization of AGMNs

The cryo-formed AGMNs were imaged using an optical microscope immediately after removal from an ultra-low temperature refrigerator. The morphologies of the needle tips marked with Alexa fluor 405 fluorescence dye, along with the FITC-mixed separating layer and the rhodamine-labeled back layer, were photographed through microscope.

### Mechanical performance testing of cryo-AGMN

The mechanical performance of the cryo AGMNs was evaluated using an Instron tensile testing machine to assess their insertion capability. The microneedle was positioned flat on a stage that had been pre-cooled in a -80 °C freezer for two hours, ensuring that the needle tips were oriented upward. The device was programmed to apply a vertical force at a constant speed of 0.5 mm/min. The testing equipment automatically recorded the force exerted on the needle tips at various displacements, generating a displacement-force curve. Data from each test were recorded and subsequently analyzed.

### Transdermal delivery of NPs using cryo-AGMNs in vivo

Male C57BL/6J mice were sourced from Vital River Lab Animal Technology Co. Prior to the experiments, abdominal hair of HFD mice (male, 14–16 weeks, 35–40 g) was removed using depilatory cream under anesthesia. Two cy5.5-labeled cryo-AGMNs were applied to the left and right flanks of the mouse abdomen under NIR808 light. Nanoparticle delivery was confirmed via in vivo imaging (IVIS Spectrum, Perkin Elmer) at specific time points (days 0, 1, 3, 7, and 14). We used image analysis software to identify microneedle application sites. Fluorescence intensity within the region of interest (ROI) was measured, and average radiant efficiency (photons/s/cm²/sr/µW) was calculated to quantify signal strength. The ROI values were adjusted by subtracting the fluorescence intensity of untreated skin from the same animal to minimize background signal interference.

### Obesity model and treatment

In experiments aimed at treating diet-induced obesity, 6-week-old male C57BL/6J mice were gave either a high-fat diet (60% kcal; D12429) or a normal chow diet (10% kcal; D12450B) for a duration of 8 weeks.

For the obesity reversion test, HFD mice were assigned into six groups; group I (low fat diet, LFD), untreated mice on a LFD; group II (HFD), mice on an HFD; group III (HFD + Cap), mice subcutaneous injected with Cap solution (Cap content: 8 mg/kg) and fed on an HFD; group IV (HFD + TmCNP): mice subcutaneous injected with TmCNP solution and fed on HFD; group V (HFD + MN@TmCNP), mice abdominally punctured with MN@TmCNP and fed on HFD; group VI (HFD + NIR&MN@TmCNP): mice on an HFD treated with MN@TmCNP under NIR irradiation (1 W/cm² at 808 nm) for 5 min. The administration was performed at the beginning of 8 weeks of HFD feeding. Microneedle treatments were administered every four weeks, while infrared irradiation was applied every three days. Groups IV-VI: Cap content: 8 mg/kg, TmCNP: 200 mg/kg.

Body weight of the sample was weighted and recorded weekly. Metabolic measurements, including GTT and ITT, were conducted at designated time points: at 18 and 22 weeks of age for animals in the obesity treatment study. Digital images of the mice were captured at the conclusion of the experiment (22 weeks of age). After an overnight fast, the mice were sacrificed and various tissues were collected for further analysis.

### TRPV1 Inhibition following MN treatment

To investigate the role of TRPV1 in mediating the metabolic effects of microneedle treatment, HFD mice were intraperitoneally injected with capsazepine (CPZ, 15 mg/kg; HY-15640, MedChemExpress) 30 min prior to each microneedle application to inhibit TRPV1 activity. Control groups received equivalent volumes of vehicle solution. At the end of the treatment period, liver and serum samples were collected to assess lipid accumulation, and scWAT was harvested for immunofluorescence staining of UCP1 expression.

### Thermal photography

Digital images were acquired using thermal infrared imaging camera. After anesthesia, the mice were positioned on a table with their abdomens facing upward, approximately 20 cm from the camera, for a 5-min video recording. The temperature of the designated area in the thermographic images was automatically measured by the camera’s built-in system.

### Histological analysis

Mice were euthanized at prescribed time points, and tissues including gel implants, liver, and fat pads, were separated. These tissues were washed in cold PBS, fixed in 10% neutral formalin for 20 h, then embedded in paraffin. Then, sections were cut to a thickness of 5 μm and subsequently stained with hematoxylin and eosin. After mounting with neutral balsam, all samples were imaged and analyzed with ImageJ software.

### Immunostaining

For immunohistochemical analysis of adipose tissue, sections were initially deparaffinized, rehydrated. Subsequently, heat-induced antigen retrieval was performed to enhance antigen detectability. After washing three times with PBS and inactivating endogenous peroxidases using a 3% H₂O₂ solution for 10 min, the sections were blocked with 5% BSA. Primary antibodies (anti-cEBPα, anti-PPARγ, and anti-TRPV1) were then incubated overnight at 4 °C. After three times with PBS, the sections were incubated with secondary antibodies at a 1:250 dilution of for 40 min at room temperature, followed by three washes in PBS. For cell nucleus staining, sections were mounted in hematoxylin solution for 3 min. All stained sections were imaged using a light microscope.

### Serum lipid measurements

To analyze the serum lipid levels, mice were fasted overnight prior to serum collection. The serum concentrations of TCHO and TG were detected via a commercial test kit (Jiancheng, Nanjing), following the product instructions.

### Liver function tests

Serum levels of aspartate amino transferase (AST) and alanine amino transferase (ALT) in mice were measured with commercial Kits (Jiancheng Bioengineering Institute) according to the manufacturer instructions.

### Statistics analysis

Results are displayed as mean ± standard deviations (SD). Significant differences between groups were calculated by Student’s unpaired *t*-test, one-way, or two-way ANOVA (Tukey’s, Skide’s and Dunett’s multiple comparison test). Non-parametric data were analyzed by the Mann–Whitney test. *p < 0.05; **p < 0.01; ***p < 0.001 considered as statistically significant. Statistical analysis was performed with GraphPad Prism 10.0 (GraphPad Software).

## Supplementary Information

Below is the link to the electronic supplementary material.

Supplementary Material 1

## Data Availability

No datasets were generated or analysed during the current study.
