# Supplementary material for: Separable cryo-microneedle patches delivery with capsaicin integrated mesoporous dopamine for obesity treatment
Source: J Nanobiotechnology. 2025 Sep 3;23:604. doi: 10.1186/s12951-025-03645-y (PMC12406480; doi:10.1186/s12951-025-03645-y)
Supplement: Supplementary file 1 — Supplementary Material 1 [file 12951_2025_3645_MOESM1_ESM.docx]

**Separable cryo-microneedle patches delivery with capsaicin integrated mesoporous dopamine for obesity treatment**

Jingjing Gan ^a^, Lingyu Sun ^b^, Wenjuan Tang ^a,^*, Yuanjin Zhao ^a,b,^*, Yan Bi ^a,^*

^a^ Department of Endocrinology, Nanjing Drum Tower Hospital, Medical School, Nanjing University, Nanjing 210002, China

^b^ State Key Laboratory of Bioelectronics, School of Biological Science and Medical Engineering, Southeast University, Nanjing 210096, China

* Corresponding author. Email: [tangwenjuan.nju@163.com](mailto:tangwenjuan.nju@163.com) (J Tang); [yjzhao@seu.edu.cn](mailto:yjzhao@seu.edu.cn) (Y Zhao); [biyan@nju.edu.cn](mailto:biyan@nju.edu.cn) (Y Bi)


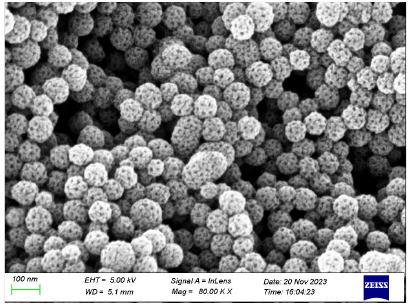


**Supplementary Fig. 1** Representative SEM micrograph of mPDA showing the morphology of its porous structure.


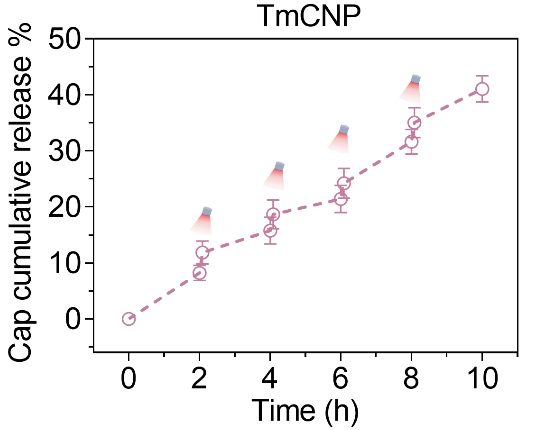


**Supplementary Fig. 2** NIR exposure triggered release of Cap from TmCNP in PBS at pH 7.4.


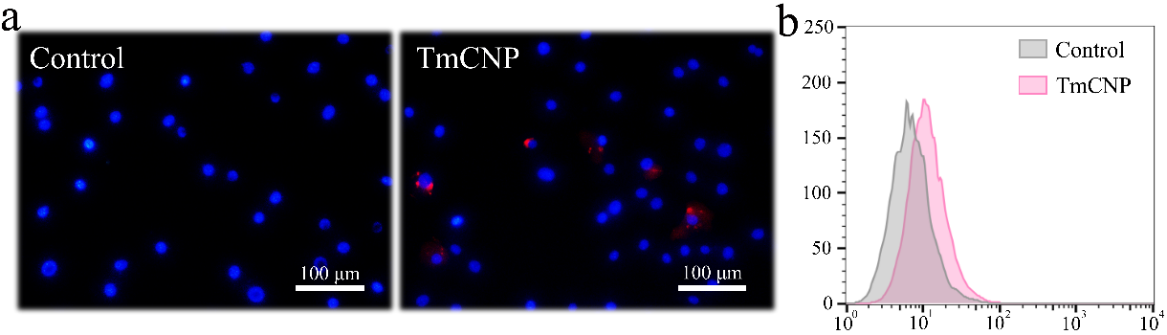


**Supplementary Fig. 3** Investigation of the uptake characteristics of TmCNPs in 3T3-Ll cells. **(a)** Confocal images showing the cellular uptake of cy5.5-labeled nanoparticles (10 μg/ml) after 6 h of incubation with 3T3-L1 cells. Cell nuclei stained blue (DAPI). Scale bar: 100 μm. **(b)** Representative flow cytometry histogram illustrating the uptake of TmCNPs by 3T3-L1 cells.


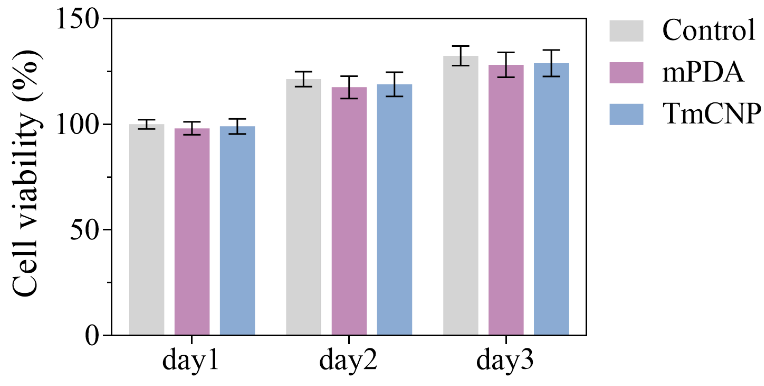


**Supplementary Fig. 4** Cytotoxic effects of NP exposure on 3T3-L1 cells over 1, 2, and 3 days.


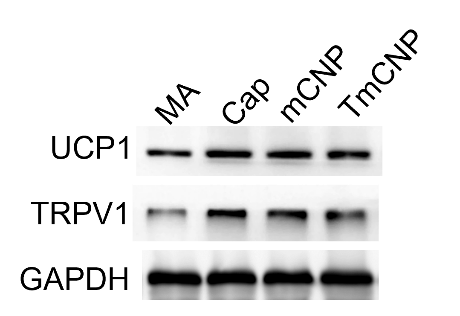


**Supplementary Fig. 5** The protein expression levels of UCP1 and TRPV1 in treated adipocytes, as determined by Western blotting.


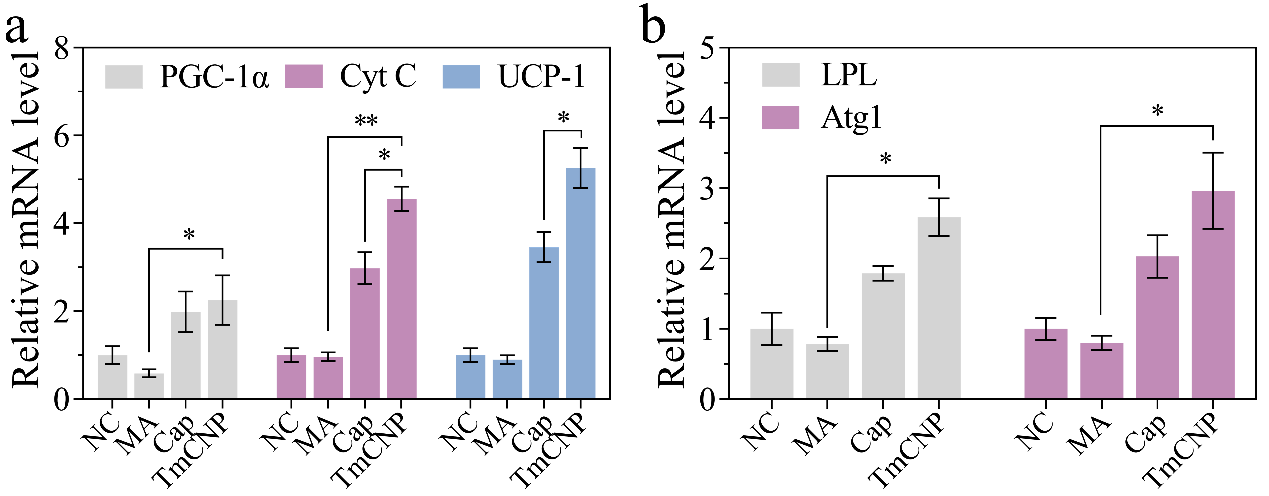


**Supplementary Fig. 6** Q-PCR analysis of mRNA levels of **(a)** mitochondria biogenesis and browning makers (PGC-1α, Cyto C, UCP1) and **(b)** lipolysis-related genes (LPL, Atgl) in mature adipocytes following various treatments.


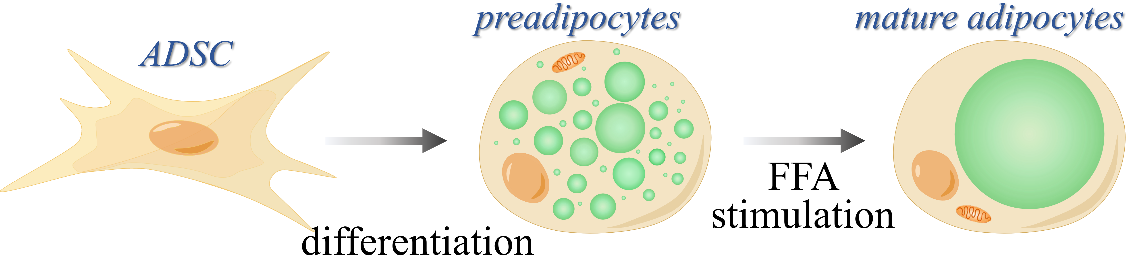


**Supplementary Fig. 7** Schematic of fatty acid uptake in vitro model using ADSCs-induced adipocytes treated with palmitic acid.


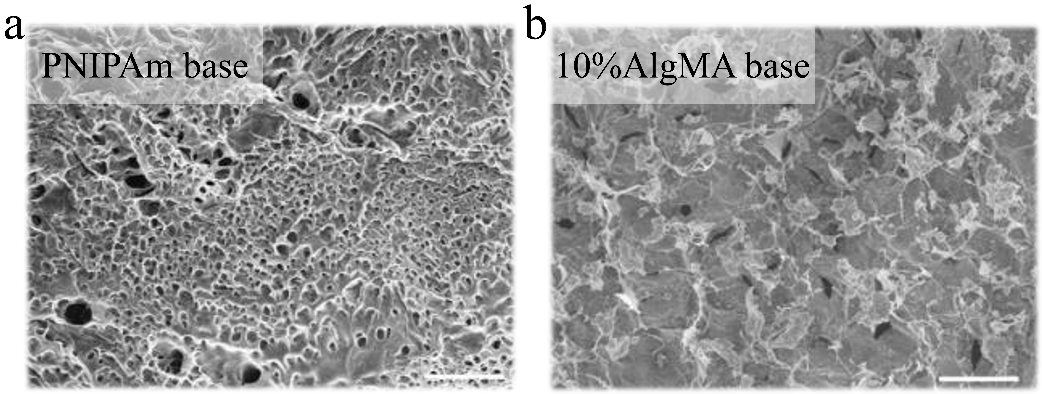


**Supplementary Fig. 8** SEM images of PNIPAm and 10% AlgMA substrates. Scale bar, 50 μm.


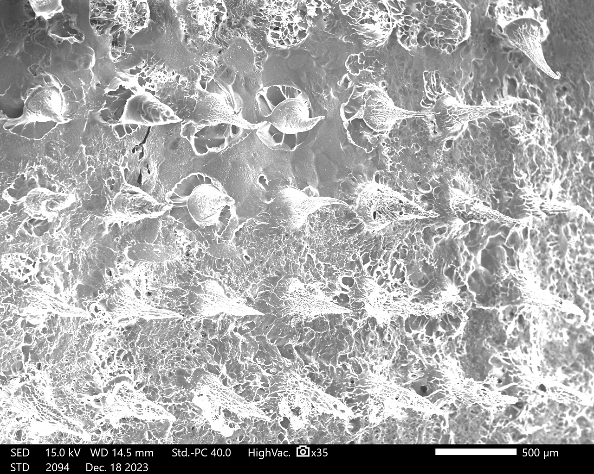

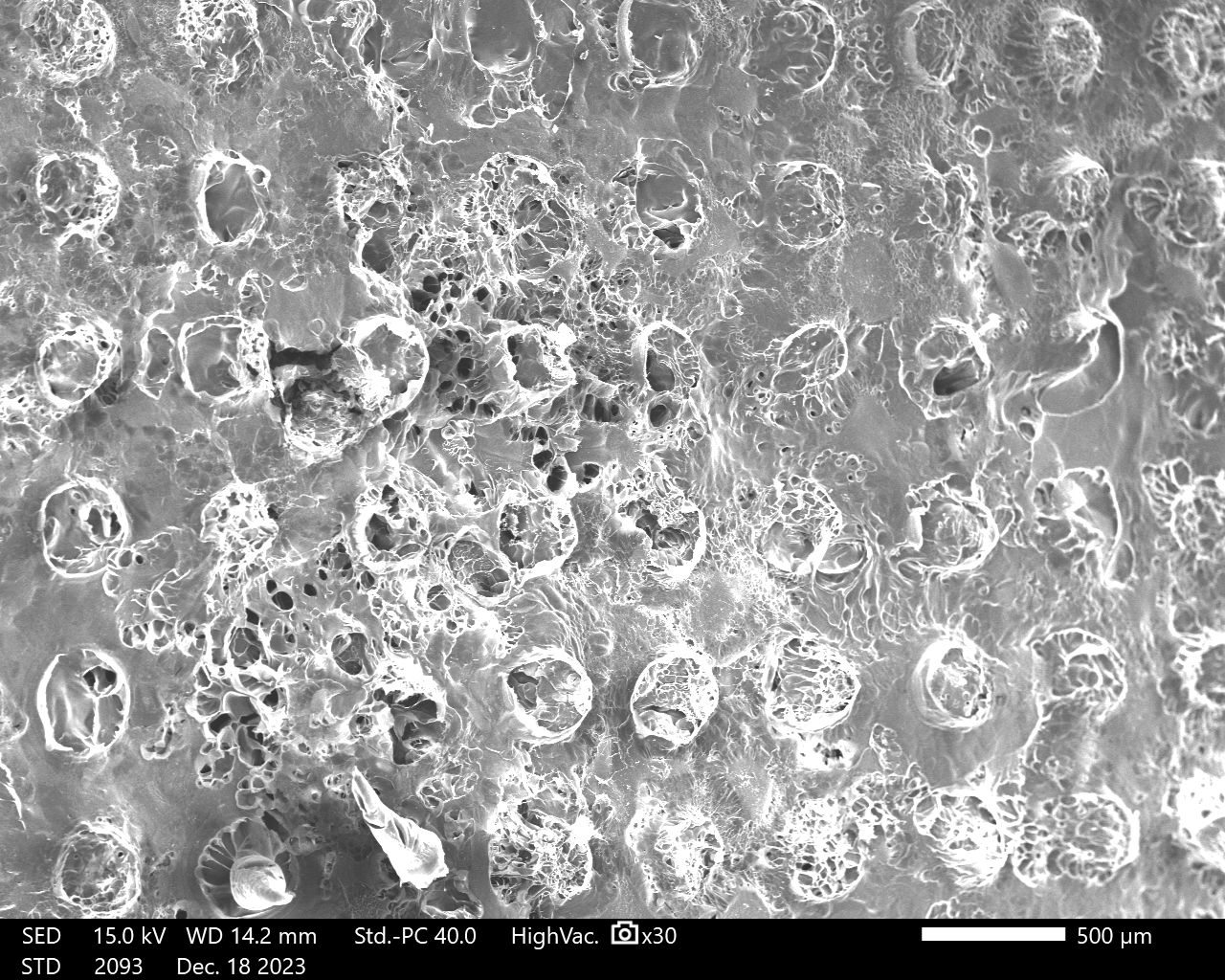

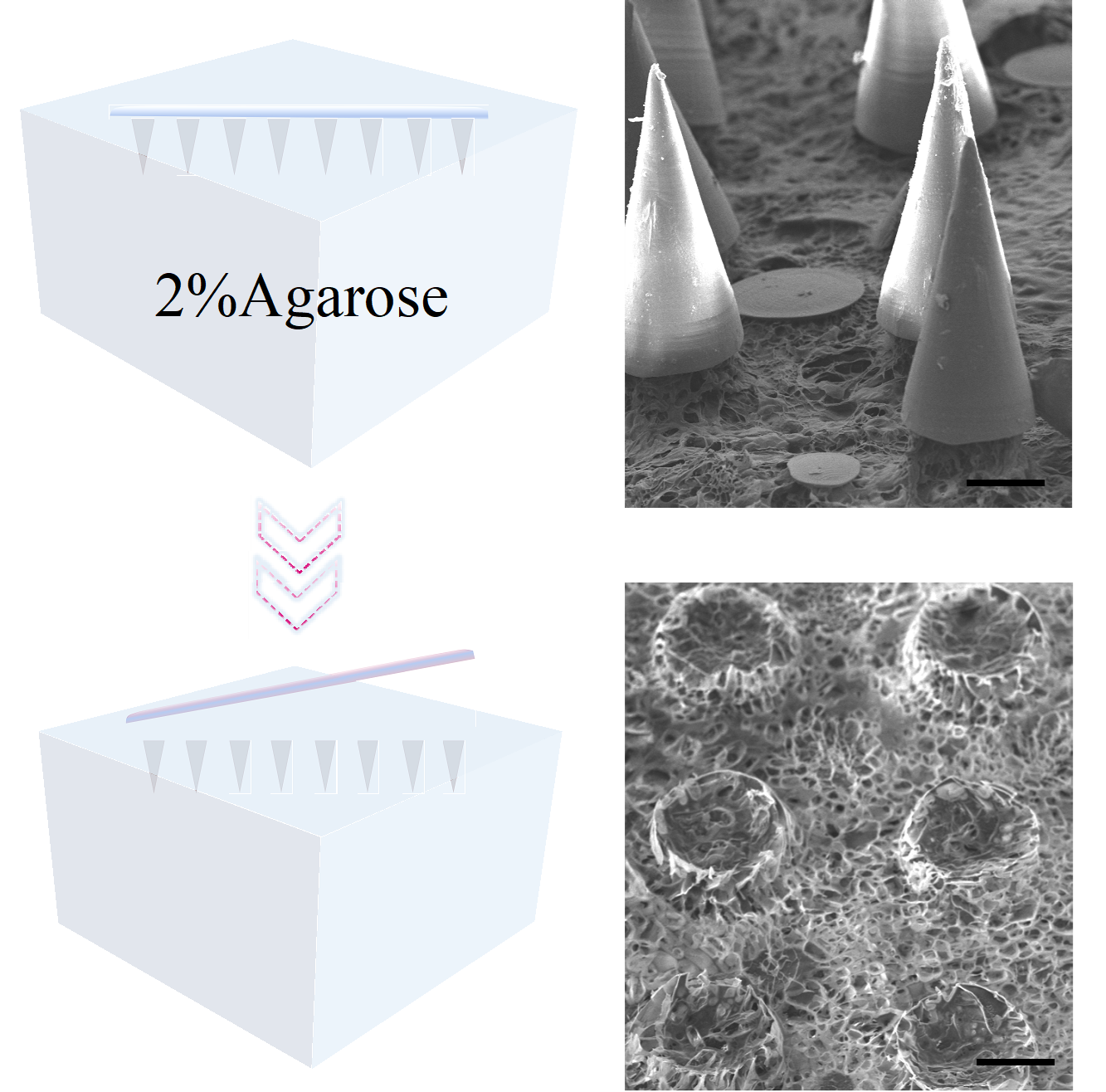


**Supplementary Fig. 9** Characterization of the separable cryo-microneedle. Schematic of 2% agarose gel for the MN tips. SEM images of the microneedle dressing before and after separation. Scale bar, 200 μm.


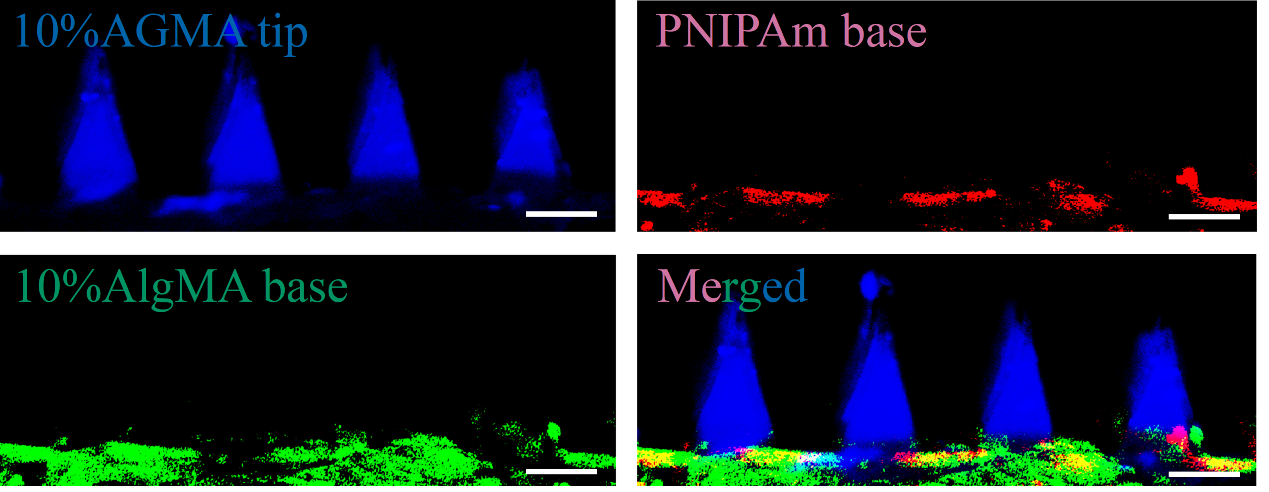


**Supplementary Fig. 10** Representative fluorescence images of AGMN patch captured using an upright fluorescence microscope. The blue fluorescence from the 10% AGMA tip mixed with Alexa Fluor 350 is distinctly observed, while the red fluorescence from PNIPAm mixed with Rhodamine B and the green fluorescence from the AlgMA base mixed with FITC are uniformly distributed beneath the microneedle.


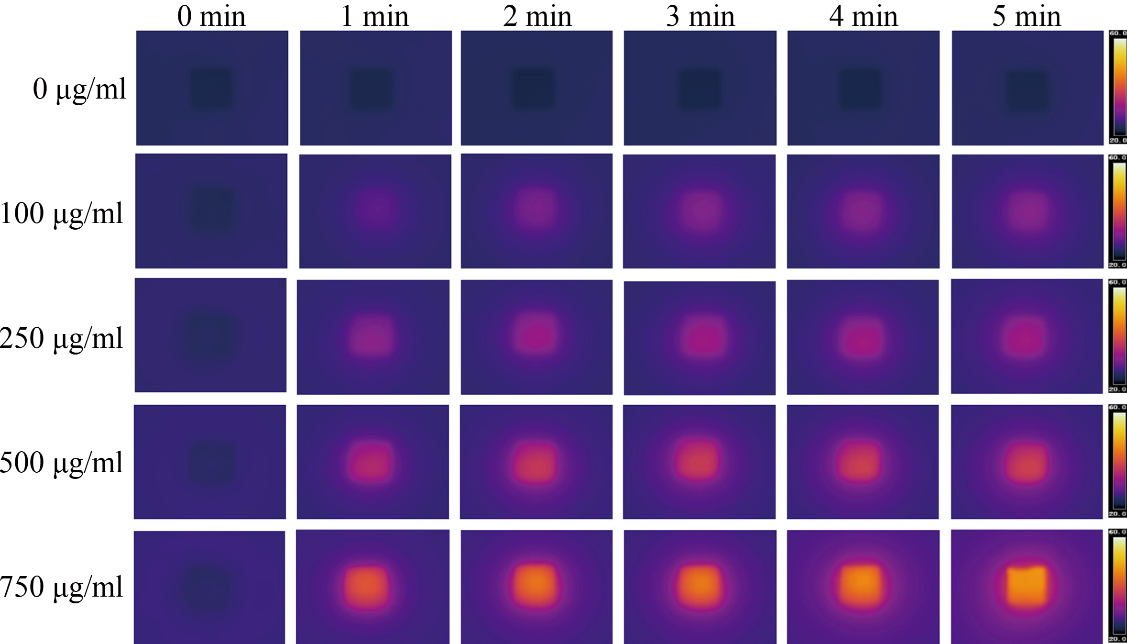


**Supplementary Fig. 11** Thermal images of AGMN at various concentrations of mPDA (0, 100, 250, 500, and 750 μg/ml) subjected to NIR at a power density of 0.75 W/cm^2^ for 5 min.


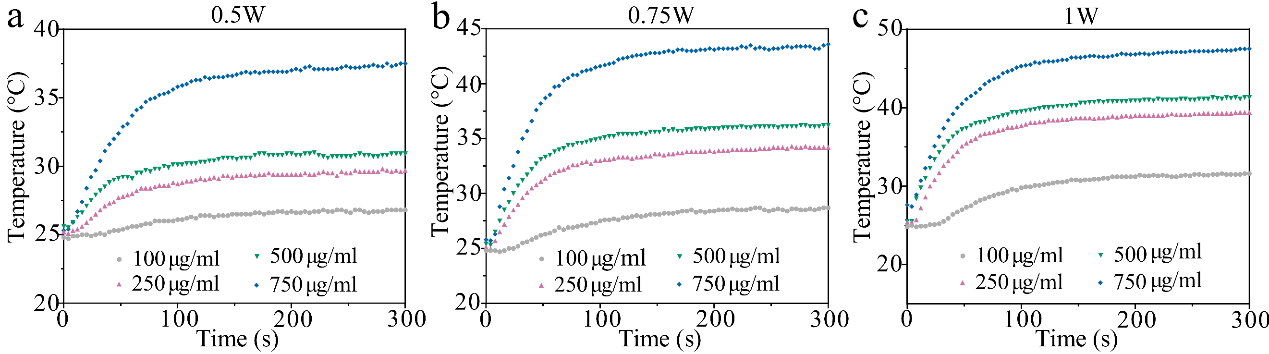


**Supplementary Fig. 12** Temperature changes of AGMN at different mPDA concentrations during irradiation with 808 nm lasers at varying intensities (0.5, 0.75, and 1.00 W/cm²) over time.


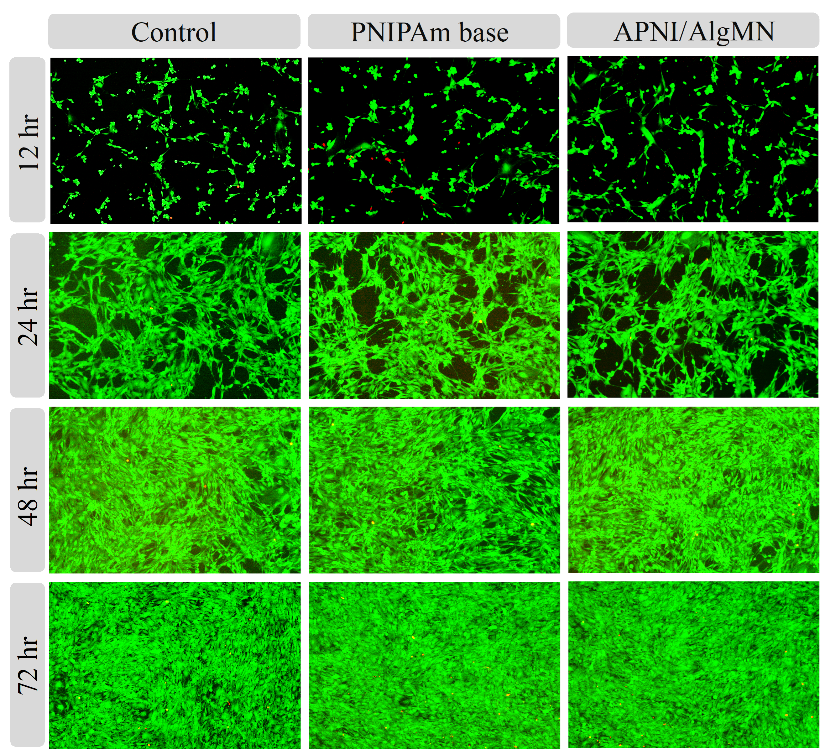


**Supplementary Fig. 13** Cell viability of AGMN in PBS over 12, 24, 48, and 72 hr.


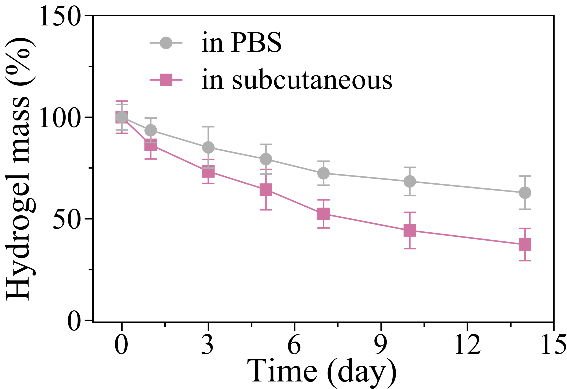


**Supplementary Fig. 14** Degradability of the needle tip material. Dry weight changes of the needle tip material in PBS solution over 10 days, illustrating in vitro degradation, with the dry weight at 0 hour set as the standard. Additionally, dry weight changes of the needle tip material beneath mouse skin over 10 days demonstrate in vivo degradation, with the dry weight at 0 hour also set as the standard.


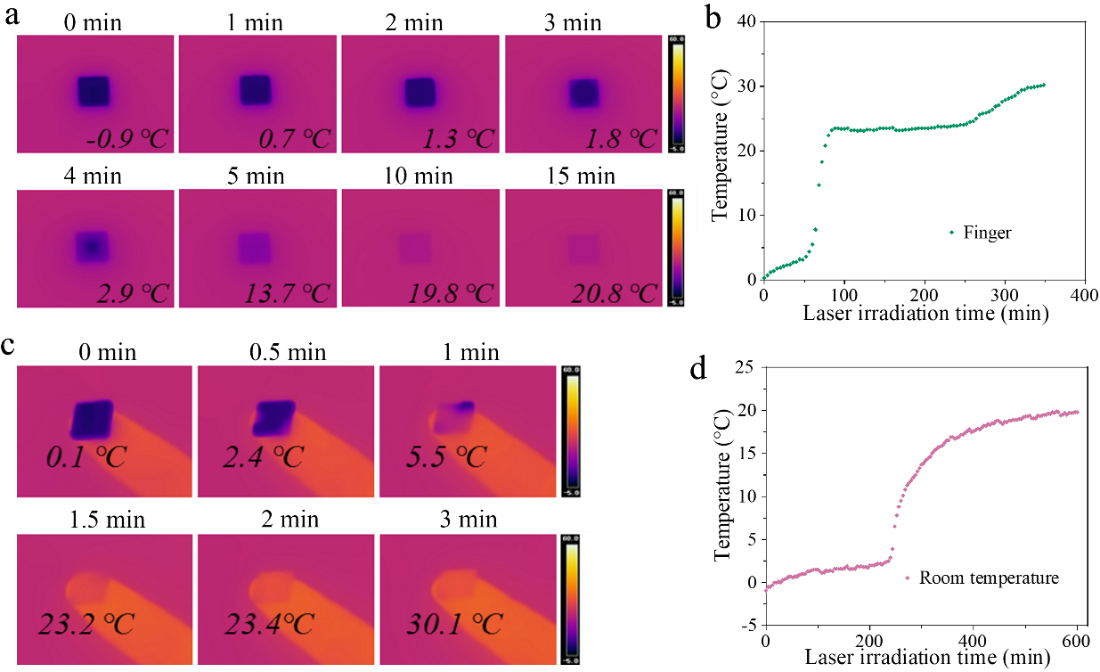


**Supplementary Fig. 15** Infrared thermal images and heating curve of MN patch (500 μg/ml mPDA) at the desktop or fingertip under room temperature.


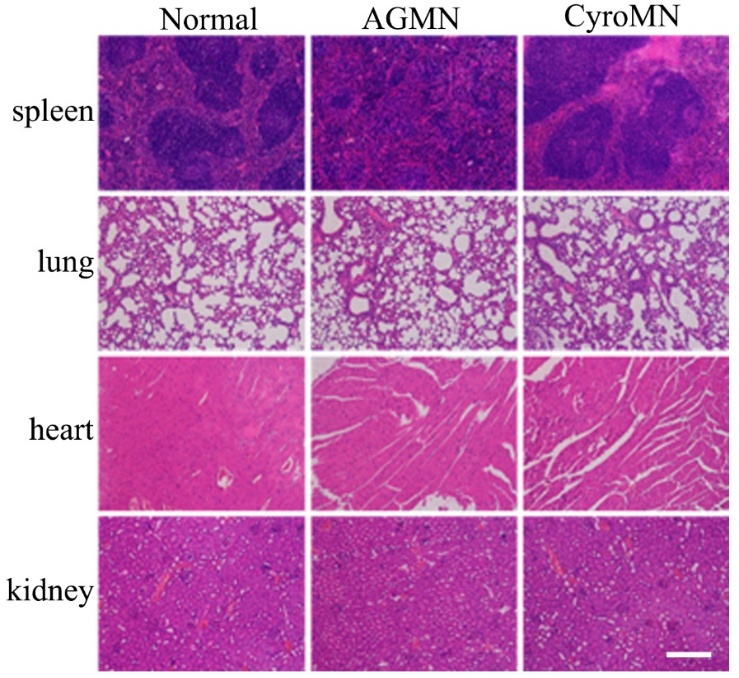


**Supplementary Fig. 16** H&E staining pictures of major tissues from animals after different treatments for 4 weeks. Scale bars: 100 μm.


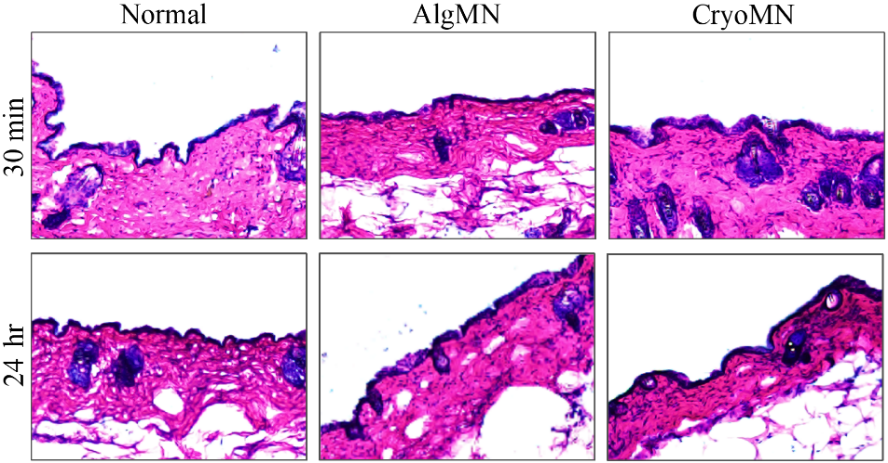


**Supplementary Fig. 17** H&E staining pictures of mice skin inserted by MNs.


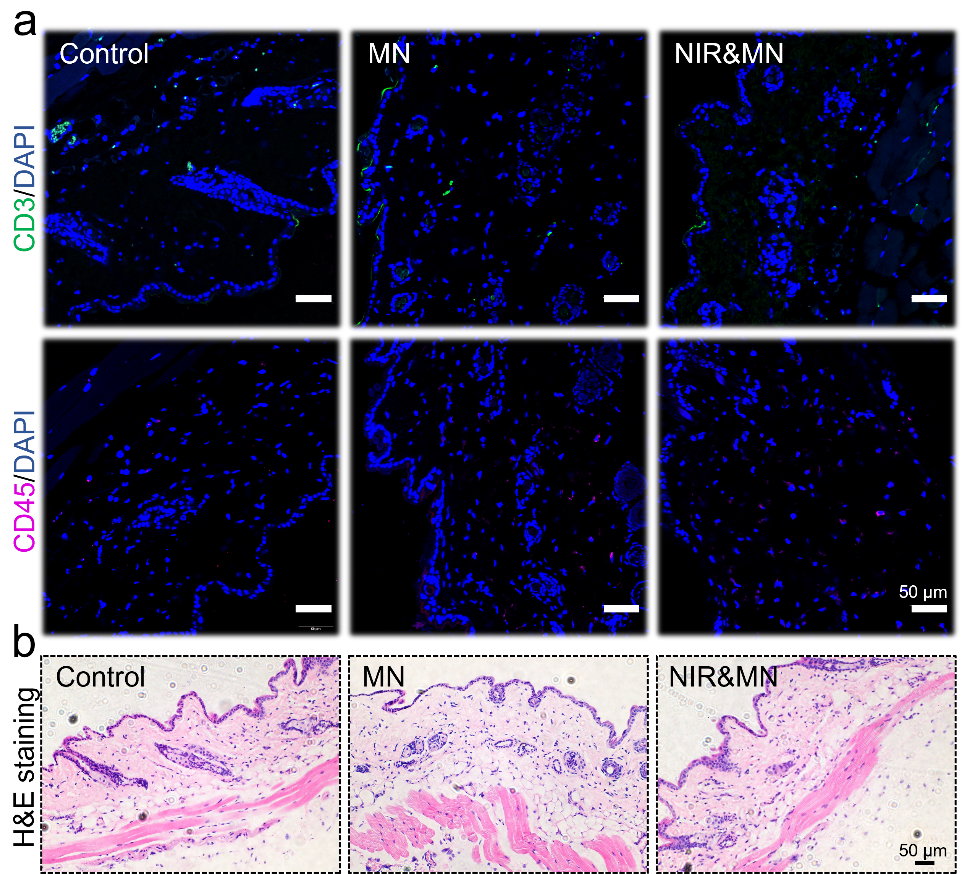


**Supplementary Fig. 18** Evaluation of immune response and biocompatibility following microneedle application. **(a)** Immunofluorescence staining for CD3 and CD45, and **(b)** HE staining, indicate no significant immune response or inflammation in mouse skin following different treatments.


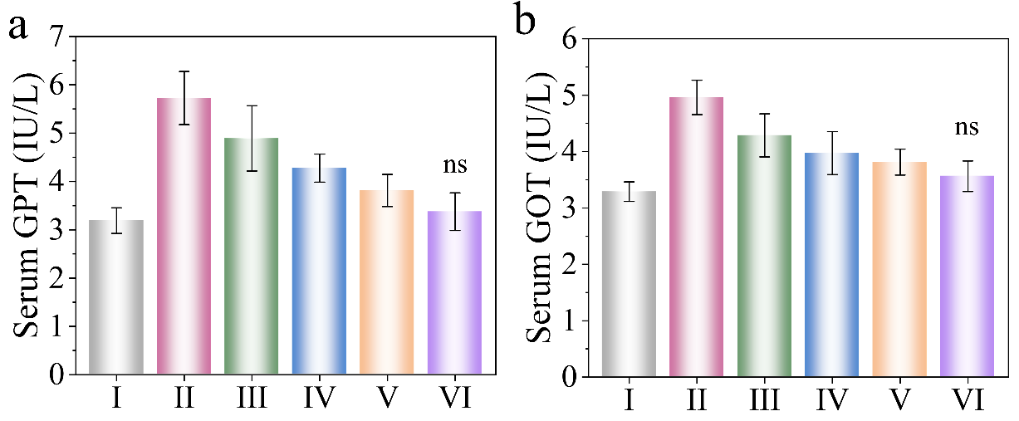


**Supplementary Fig. 19** Quantification of serum glutamic pyruvic transaminase (GPT) and aspartic aminotransferase (GOT) level. ns: no significant.


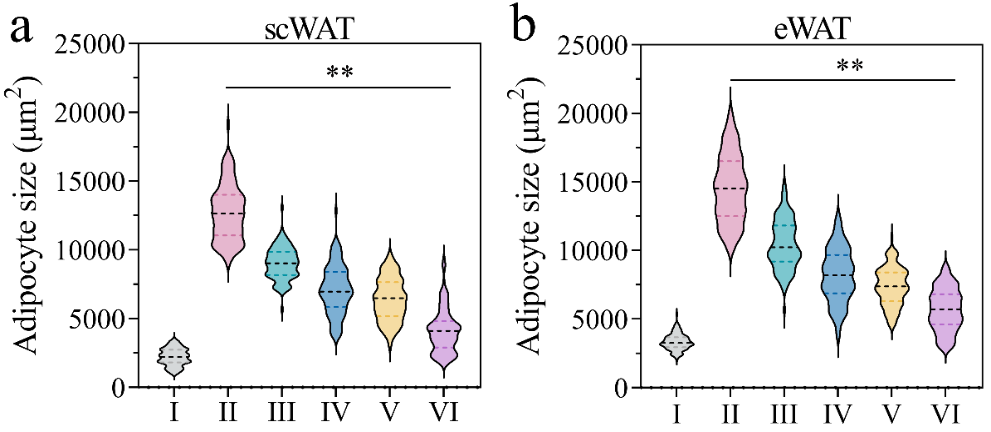


**Supplementary Fig. 20** Quantification of adipocyte sizes in scWAT and eWAT. ***p*<0.01


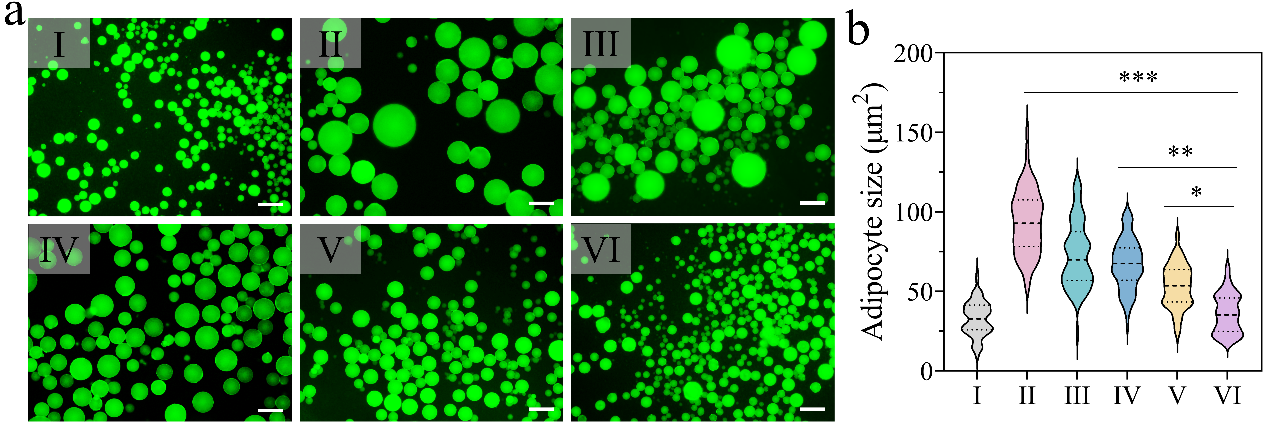


**Supplementary Fig. 21** The diameters of lipid droplets in adipose tissue were evaluated after treatment across different groups. Adipocytes were stained with BODIPY and examined using confocal microscopy, with scale bars indicating 100 μm. Corresponding statistical results for adipocyte volume are presented, with significance levels indicated as ****p* < 0.001, ***p* < 0.01, and **p* < 0.05.


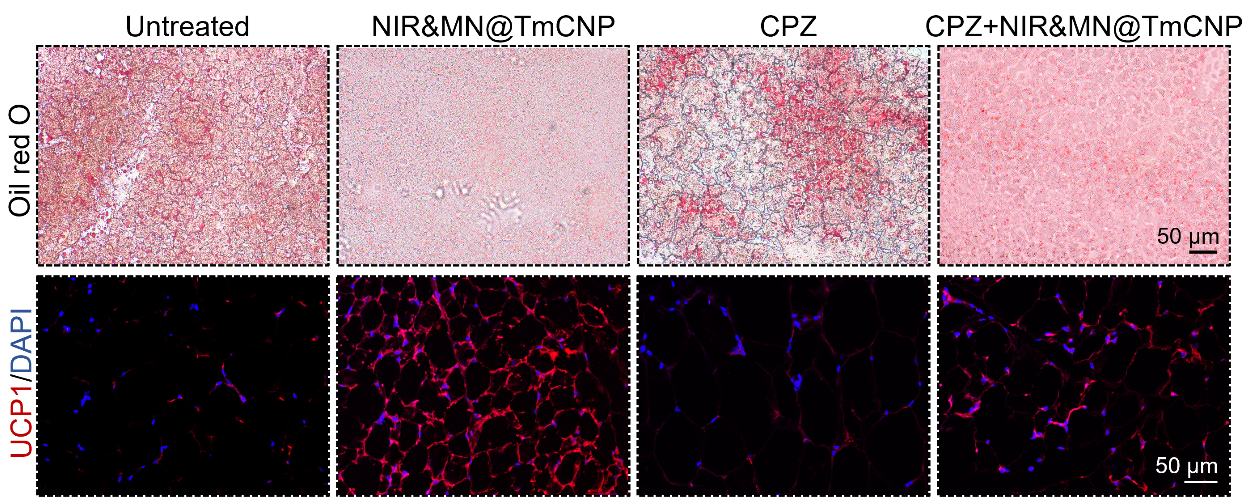


**Supplementary Fig. 22** Oil Red O staining images of the liver and immunofluorescence images of UCP1 in mice from the control, NIR&MN@TmCNP, CPZ, and CPZ+NIR&MN@TmCNP groups.


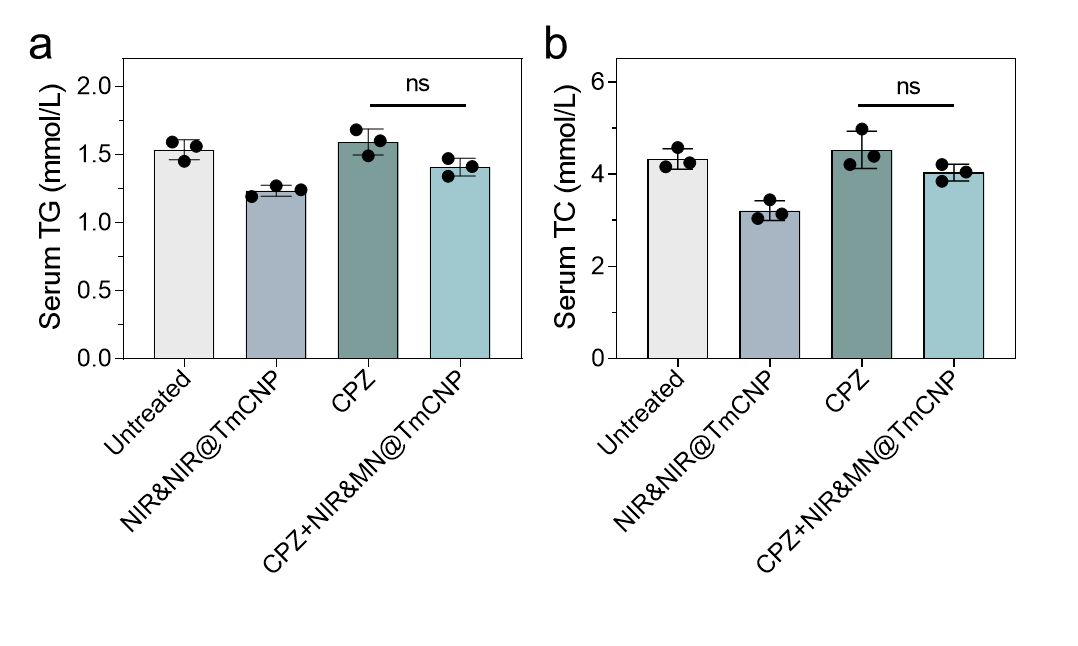


**Supplementary Fig. 23** Quantification of serum TG and TC level. ns: no significant.


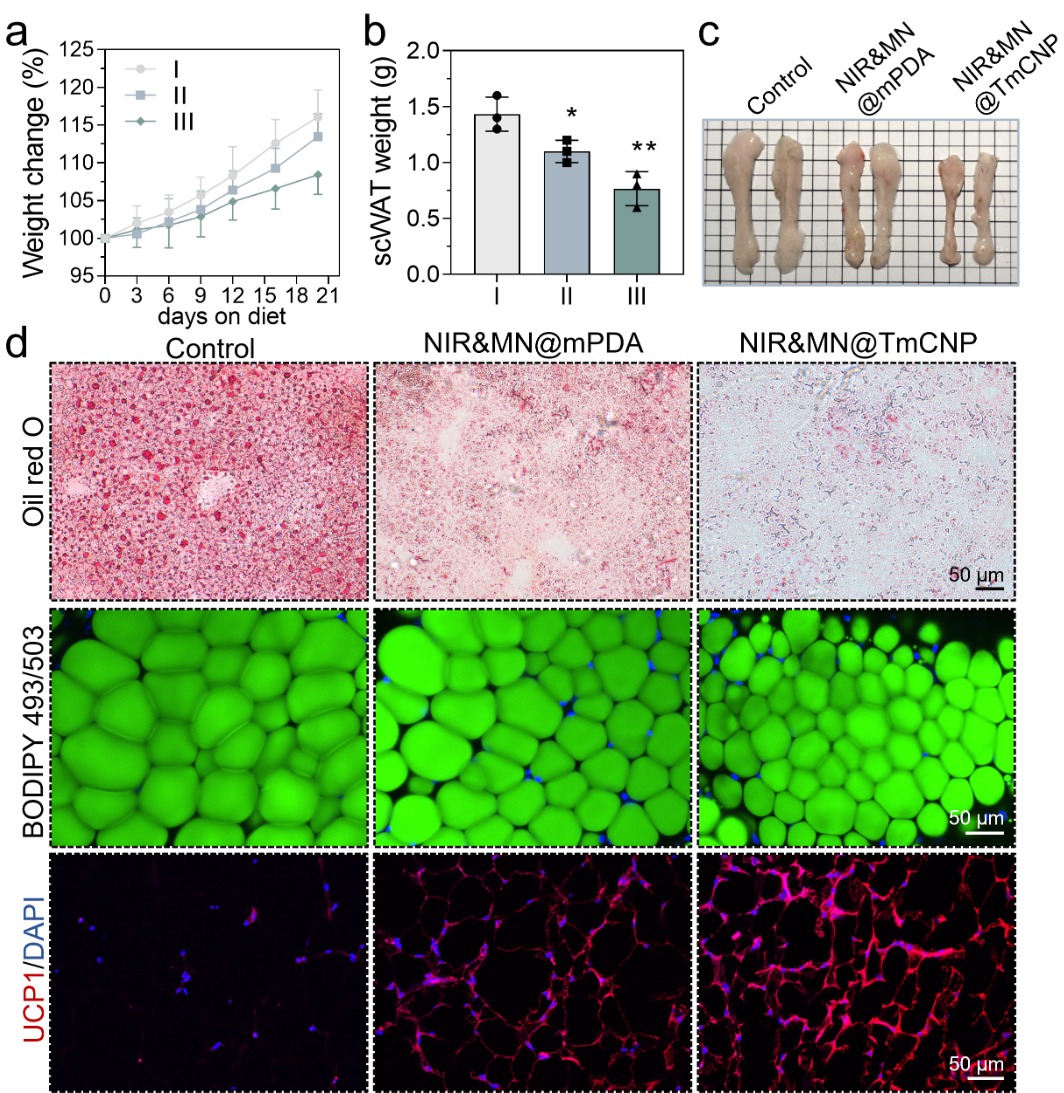


**Supplementary Fig. 24 (a)** Body weight changes in mice from the control, NIR&MN@mPDA, and NIR&MN@TmCNP groups; **(b)** Weight and **(c)** representative images of scWAT; **(d)** Oil Red O staining of the liver, BODIPY fluorescence staining of lipid droplets in abdominal adipose tissue, immunofluorescence staining for UCP1.

**Table S1.** Murine primer sequences used in Q-PCR analysis.

| No. | Gene | Sequence (5’- 3’) |
| --- | --- | --- |
| 1 | mus-β-actin Forward  mus-β-actin Reverse | GGTGTGATGGTGGGAATGGG  ACGGTTGGCCTTAGGGTTCAG |
| 2 | mus-PPARγ Forward  mus-PPARγ Reverse | GACCTTAATTGTCGCATCCAT  CGGGAAGGACTTTATGTATGA |
| 3 | mus-cEBPα Forward  mus-cEBPα Reverse | CGGACTTGGTGCGTCTAAG  CATTGGAGCGGTGAGTTTG |
| 4 | mus-TRPV1-Forward  mus-TRPV1-Reverse | GTTTACCTCGTCCACCCTGA  AGAGAGCCATCACCATCCTG |
| 5 | mus-mtDNA-Forward  mus-mtDNA-Reverse | CGCCCTAACAACTATTATCTTCC  GACCGTTTGTTTGTTGTTGAAAA |
| 6 | mus-B2M Forward  mus-B2M Reverse | AGATGAGTATGCCTGCCGTG  TCATCCAATCCAAATGCGGC |
| 7 | mus-PGC-1α-Forward  mus-PGC-1α-Reverse | AAACTTGCTAGCGGTTCTCAC  GGCAATCCGTCTTCATCCAC |
| 8 | mus-Cytc-Forward  mus-Cytc-Reward | AAATCTCCACGGTCTGTTCGG  GGGTATCCTCTCCCCAGGTG |
| 9 | mus-UCP1-Forward  mus-UCP1-Reward | GGCCCTTGTAAACAACAAAATAC  GGCAACAAGAGCTGACAGTAAAT |
| 10 | mus-LPL-Forward  mus-LPL-Reward | GCCCAGCAACATTATCCAGT  GGTCAGACTTCCTGCTACGC |
| 11 | mus-ATGL-Forward  mus-ATGL-Reward | CAACGCCACTCACATCTACGG  GGACACCTCAATAATGTTGGCAC |
